# Benzotriazole‐Functionalized Ionic Liquid and Superwettability‐Assisted Transfer Enable Air‐Stable, Large‐Area Copper Nanowires‐Based Flexible Transparent Electrodes

**DOI:** 10.1002/advs.202515330

**Published:** 2025-11-03

**Authors:** Bin Hou, Kaiyan Wu, Yuying Deng, Shuo Wang, Wei Wang, Hongqin Wang, Dong Ding, Chuao Ma, Honglei Fan, Hongliang Liu, Lei Jiang

**Affiliations:** ^1^ Shandong Laboratory of Advanced Materials and Green Manufacturing at Yantai Yantai 264006 China; ^2^ School of Chemistry and Chemical Engineering Yantai University Yantai 264005 China; ^3^ Key Laboratory of Bio‐inspired Materials and Interfacial Science Technical Institute of Physics and Chemistry Chinese Academy of Sciences Beijing 100190 China

**Keywords:** copper nanowires, flexible optoelectronics, flexible transparent electrodes, ionic liquid, superwettability‐assisted transfer

## Abstract

Copper nanowires (CuNWs) are promising for flexible transparent electrodes but suffer from lack of effective strategies to inhibit the oxidation‐induced conductivity degradation, especially during large‐area electrode preparation. Herein, a benzotriazole‐functionalized ionic liquid ([BTAMMIM]TFSI) is introduced as an antioxidant layer to protect the CuNWs networks. Remarkably, the sheet resistance of the protected electrodes increases by only 0.54% compared to bare CuNWs after 60‐day air exposure. Density functional theory (DFT) calculations and experiments reveal that the benzotriazole‐functionalized cations and [TFSI] anions synergistically coordinate with copper, enabling exceptional oxidation resistance. By integrating with superwettability‐assisted interfacial transfer strategy, large‐area CuNWs@[BTAMMIM]TFSI composite electrodes (40 × 25 cm^2^) are fabricated with 37.2 Ω sq^−1^ sheet resistance and 88.2% transmittance (550 nm). The electrodes maintain performance under acidic/alkaline conditions (pH 3/13) and high humidity (85% RH) at 85 °C. A demonstrated flexible smart window exhibits high transmittance modulation (6.1%–68.2%), fast response (< 0.2 s) and long‐term stability, highlighting their potential in flexible optoelectronics.

## Graphical abstract

Benzotriazole‐functionalized ionic liquids, which exhibit excellent oxidation‐resistant properties for copper nanowires‐based flexible transparent electrode owing to the anion‐cation synergistic protective effect, are integrated with the superwettability‐assisted transfer, a strategy for the spontaneous transfer of copper nanowires from air–water interface to substrates driven by surface tension difference, to afford air‐stable, large‐area copper nanowires‐based flexible transparent electrodes.

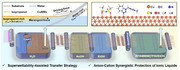

## 1 Introduction

Flexible transparent electrodes have received more and more attention in recent years due to their wide‐spread application in many optoelectronic devices such as solar cells,^[^ ^1^, ^2^ ^]^ light‐emitting diodes,^[^ ^3^ ^]^ touch screen^[^ ^4^, ^5^ ^]^ and wearable electronics. ^[^ ^6^ ^]^ Due to the high transmittance and low sheet resistances, indium tin oxide (ITO) is the dominant material for constructing transparent electrodes. However, the application of ITO is severely limited because of brittleness, scarcity of indium resources and high‐cost processing conditions.^[^ ^7^ ^]^ Therefore, many materials including carbon nanotubes,^[^ ^8^ ^]^ graphene,^[^ ^9^ ^]^ metal nanowires^[^ ^10^, ^11^ ^]^ as well as conducting polymers^[^ ^12^ ^]^ have been developed to substitute ITO to construct transparent electrodes. Among these materials, metal nanowires are particularly promising owing to their intrinsically high electrical conductivity, wherein some conductive films of sliver nanowires have exhibited better conductivities and transmittance than those of ITO.^[^ ^13^ ^]^ Compared with silver, copper has the similar electrical resistivity. What's more, copper is 1000 times more abundant and 100 times less expensive.^[^ ^14^, ^15^ ^]^ Therefore, it will enormously reduce the cost while using copper nanowires (CuNWs) to fabricate flexible transparent electrodes.

The application of CuNWs‐based electrodes is severely restricted by long‐term stability, because CuNWs are easy to be oxidized when exposed to air. In the past few years, many efforts have been made to develop antioxidant layers for CuNWs‐based electrodes. Common strategies involving metallic oxide coating,^[^ ^16^, ^17^ ^]^ metal coating,^[^ ^18^, ^19^ ^]^ and graphene covering,^[^ ^20^ ^]^ can effectively protect the CuNWs from being oxidated. Despite the oxidation‐resistant property, there are some issues with these methods, especially during large‐area electrode preparation. For example, methods like metallic oxide and metal coating usually make the conductive CuNWs layer thicker and rougher, thus increasing the risk of shunting or shorting during application.^[^ ^21^, ^22^ ^]^ Graphene covering is often subjected to harsh manufacturing conditions and lattice defects of the graphene layer,^[^ ^23^ ^]^ which may restrict the large‐area preparation and oxidation‐resistant properties of the corresponding flexible transparent electrodes to some extent. As an effective, inexpensive and easy‐to‐design approach, organic protection layers may be one of the best choices for passivating the CuNWs networks owing to their little influence on the surface morphology and transparency of the corresponding flexible transparent electrodes, as well as their suitability for large‐area protection. In fact, organic compounds like alkylamines, alkylthiols and benzotriazole have been already used as an oxidation‐resistant layer to protect the CuNWs from oxidation.^[^ ^24^, ^25^ ^]^ Thanks to the good coordination effect of heteroatoms (N or S), the organic compounds can strongly bind on CuNW surfaces, which provides excellent oxidation protection for CuNWs. However, owing to the insulation of the organic compounds, these strategies are easy to lead to an increase in the sheet resistance of the CuNW networks.^[^ ^25^, ^26^ ^]^ Recently, the study of ionic compounds as an oxidation‐resistant layer for CuNWs flexible transparent electrodes has received great attention.^[^ ^27^ ^]^ The surface coordination layer formed by the interaction between copper and formate anions can efficiently inhibit the oxidation of the copper without affecting the electrical conductivities of the CuNWs networks. Nevertheless, further use of alkanethiol to treat the CuNWs networks is essential to protect the defect sites that are not protected by the passivation layer, which makes the processing complicated and difficult for large‐area preparation. Therefore, it is meaningful to develop novel ionic compounds which can furnish CuNWs with multiple interactions to inhibit the oxidation of CuNWs for easy fabrication of large‐area, air‐stable CuNWs electrodes.

**Figure 1:**
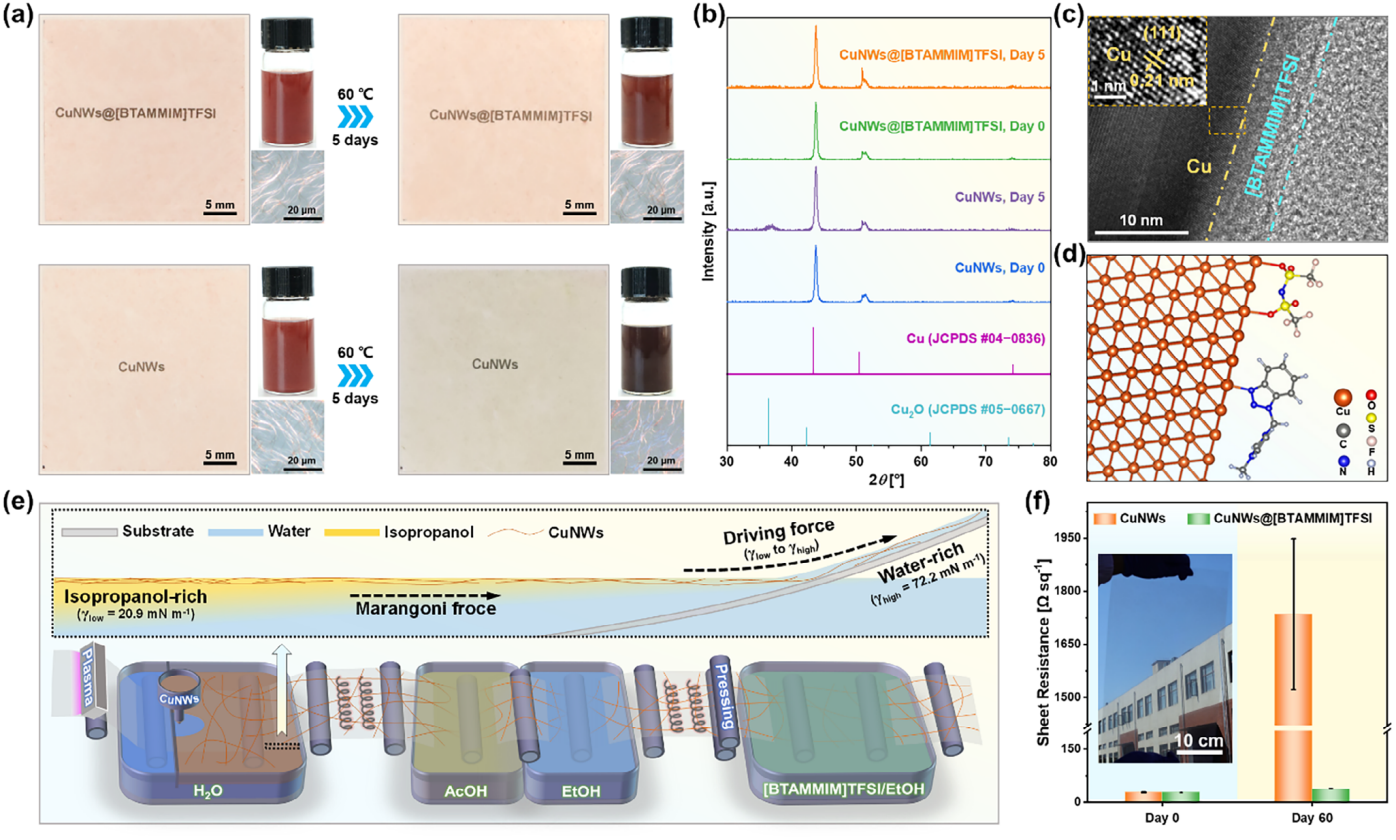
a) Change of the optical and microscopic images of CuNWs and CuNWs@[BTAMMIM]TFSI composite in film and solution states after heating at 60 °C for 5 days. The color of CuNWs without [BTAMMIM]TFSI protection changed obviously from orange to blue in the microscopic images. b) Change of the XRD pattern of CuNWs and CuNWs@[BTAMMIM]TFSI composite films after heating at 60 °C for 5 days with the PDF card of copper (JCPDS #04−0836) and cuprite (JCPDS #05−0667) as reference. c) HRTEM image of CuNWs@[BTAMMIM]TFSI composite. d) Optimized structure and coordination site of the adsorbed [BTAMMIM]TFSI on copper obtained by DFT calculations. e) Schematic illustration of superwettability‐assisted transfer strategy combined with ionic liquids treatment for large‐area preparation of CuNWs@[BTAMMIM]TFSI composite flexible transparent electrodes. f) Change in the sheet resistance of CuNWs electrodes and CuNWs@[BTAMMIM]TFSI composite electrodes after exposure in air for 60 days. Inset: An optical image of a large‐area CuNWs@[BTAMMIM]TFSI composite electrode.

Ionic liquids, as a class of representative ionic organic compounds, has the merit of easy to alter anions and cations, which may afford multiple interactions with CuNWs through adjustment of anions and side groups on the cations, thus expected to result in excellent protection for CuNWs networks without additional processing. In our previous study, we have revealed the coordination between copper and bis(trifluoromethanesulfonyl)imide [TFSI] anions of imidazolium‐base ionic liquids provides the ionogel/copper grid composites with good stability.^[^ ^28^ ^]^ Herein, five types of [TFSI]‐based ionic liquids with different cations are selected to evaluate the oxidation‐resistant protection for CuNWs networks. Wherein, the ionic liquid of 1‐((1H‐benzo[*d*][1,2,3]triazol‐1‐yl)methyl)‐3‐methylimidazolium bis((trifluoromethyl)sulfonyl)imide ([BTAMMIM]TFSI) exhibits the excellent oxidation‐resistant property for CuNWs electrodes. As illustrated in **Figure** **1**a, optical and microscopic images show that the CuNWs treated with [BTAMMIM]TFSI possess better oxidation resistance than bare CuNWs in both film and solution states at 60 °C for 5 days. The X‐ray diffraction (XRD) pattern (Figure 1b) demonstrates the same result that the (111) diffraction peak of Cu_2_O obviously emerges at 2*θ* of 36.4° for CuNWs film after heating at 60 °C for 5 days, while the CuNWs@[BTAMMIM]TFSI composite film has no diffraction peaks of Cu_2_O under the same conditions. The existence of [BTAMMIM]TFSI layer on the surface of CuNWs can be clearly observed by high‐resolution transmission electron microscope (HRTEM) image (Figure 1c). Quartz crystal microbalance (QCM) measurements and density functional theory (DFT) calculations reveal that [BTAMMIM]TFSI can interact with CuNWs through the anion‐cation synergistic coordination of the N at benzotriazole unit and O at [TFSI] anions (Figure 1d), thus affording a high oxidation‐resistant stability for the CuNWs@[BTAMMIM]TFSI composite films. To achieve the large‐area preparation of CuNWs composite electrodes, the superwettability‐assisted transfer strategy, an effective solution‐processable method which has been proved to be feasible in constructing nanomaterial thin films,^[^ ^29^, ^30^, ^31^ ^]^ is carefully studied for the fabrication of CuNWs networks (Figure 1e). Under the driving force of surface tension (*γ*) difference between dispersive solvent (isopropanol containing 0.09 mg mL^−1^ PVP, 20.9 mN m^−1^, Figure S4a, Supporting Information) and water (72.2 mN m^−1^, Figure S4b, Supporting Information), CuNWs are spontaneously transferred from air‐water interface to substrates. Combined with the processing of [BTAMMIM]TFSI (Figure 1e), a large‐area CuNWs@[BTAMMIM]TFSI composite flexible transparent electrodes with the size of 40 cm in length and 25 cm in width (Figure 1f) is successfully fabricated. The composite electrodes show an extremely low sheet resistance of 37.2 Ω sq^−1^ and a high transparency of 88.2% at 550 nm, which can remain good conductivities after 60 days (Figure 1f). Finally, a flexible smart window, which presents high transmittance variation, fast response and good durability, is constructed with the CuNWs@[BTAMMIM]TFSI composite flexible transparent electrodes, demonstrating the promising applications of the composite flexible transparent electrodes in the flexible optoelectronics fields.

## 2 Results and Discussion

### 2.1 Synthesis and Characterization of CuNWs

The CuNWs used in this work were prepared according to the reported literatures,^[^ ^32^ ^]^ followed by a multiphase separation and purification step to remove the copper nanoparticles and impurities in the course of synthesis.^[^ ^33^ ^]^ The curve in Figure S5 (Supporting Information) shows the XRD pattern of the CuNWs after purification. The (111), (200) and (220) diffraction peaks at 2*θ* of 43.3, 51.0 and 73.7°, respectively, are matched well to the cubic phase of Cu (JCPDS #04−0836), which suggests the successful preparation of CuNWs. The high intensity ratio of (111) and (200) peaks indicates that the copper nanoparticles have been removed effectively.^[^ ^33^ ^]^ In addition, the UV‐visible absorption spectrum (Figure S6, Supporting Information) of CuNWs in isopropanol displays an absorption peak at 567 nm, which is attributed to the plasma excitation in CuNWs.^[^ ^34^ ^]^ All of these results demonstrate that the CuNWs have been synthesized with high purity. Subsequently, scanning electron microscope (SEM) was applied to estimate the diameter and length of the CuNWs. As shown in Figure S7 (Supporting Information), the average diameter and length statistically obtained from 200 random CuNWs are 48 nm and 100 µm, respectively. Considering the high aspect ratios (length/diameter > 2000) of the CuNWs, the corresponding flexible transparent electrodes are expected to exhibit excellent optical and electrical performances.

### 2.2 Oxidation‐Resistant Studies of Ionic Liquids for CuNWs

**Figure 2:**
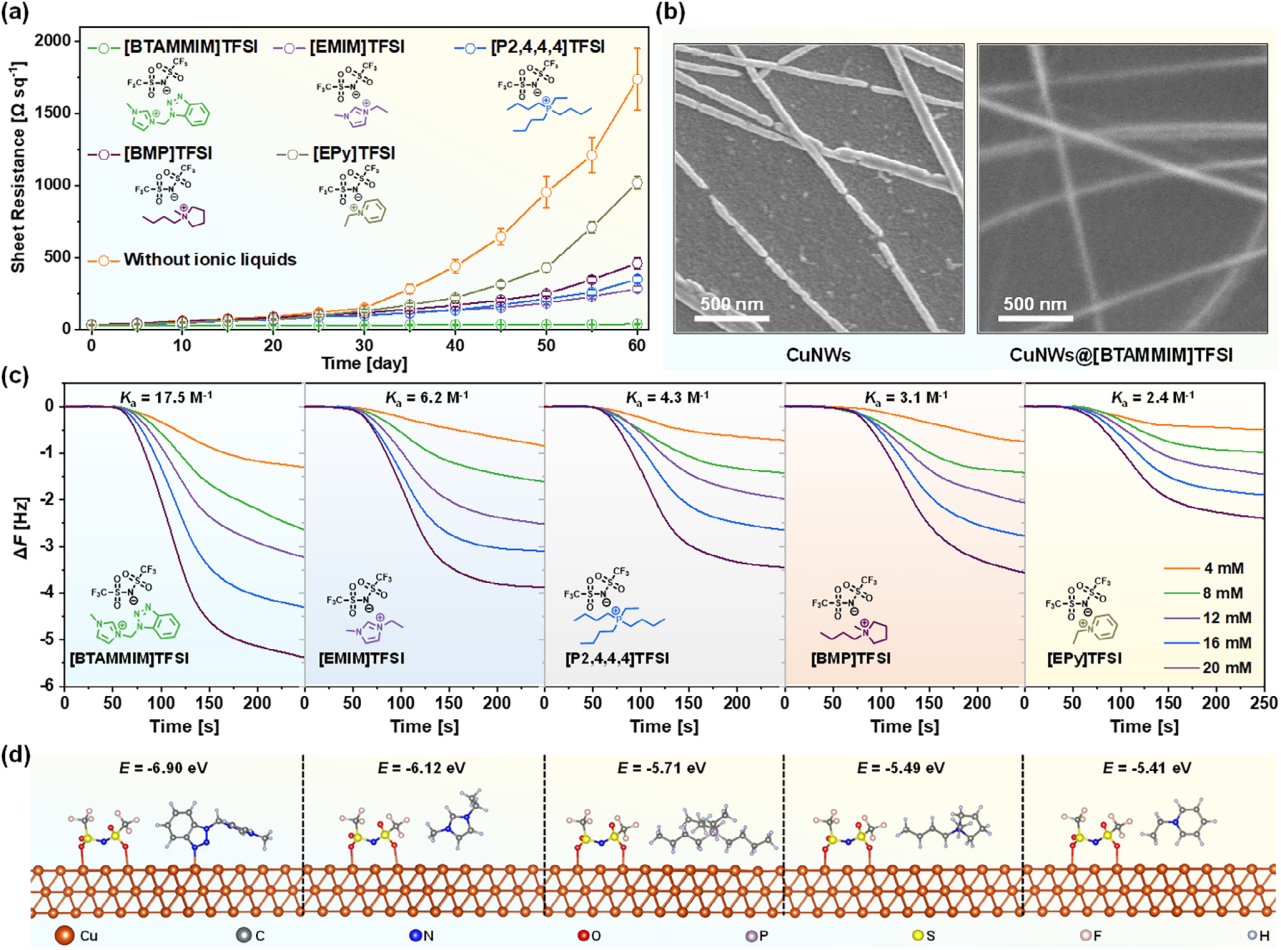
a) Changes in the sheet resistance of bare and different ionic liquids‐protected CuNWs electrodes exposed in air for 60 days. b) SEM images of CuNWs electrodes and CuNWs@[BTAMMIM]TFSI composite electrodes after exposure in air for 60 days. c) Typical curves of frequency changes after injecting 4, 8, 12, 16, and 20 mm of different ionic liquids solutions into the QCM chamber with Cu‐coated QCM sensors. d) Side views of optimized structures and coordination sites of different ionic liquids on copper obtained by DFT calculations.

On the basis of the high‐quality CuNWs, the key role of ionic liquids to protect the CuNWs from being oxidated are investigated. According to our previous study, ionic liquids with [TFSI] anions show good oxidation‐resistant property for copper grid.^[^ ^28^ ^]^ Therefore, five types of [TFSI]‐based ionic liquids with different cations, [BTAMMIM]TFSI, 1‐ethyl‐3‐methylimidazolium bis(trifluoromethylsulfonyl)imide ([EMIM]TFSI), ethyltributylphosphonium bis((trifluoromethyl)sulfonyl)imide ([P2,4,4,4]TFSI), 1‐butyl‐1‐methyl pyrrollidinium bis(trifluoromethylsulfonyl)imide ([BMP]TFSI) and *N*‐ethylpyridinium bis((trifluoromethyl)sulfonyl)imide ([EPy]TFSI), are chosen to evaluate their oxidation‐resistant properties for CuNWs films. It is worth noting that the chemical structure of [BTAMMIM]TFSI can be regarded as a combination of [EMIM]TFSI and benzotriazole group via a methylene group. The unique structure may afford multiple interactions with CuNWs, which is expected to endow the [BTAMMIM]TFSI with the best oxidation‐resistant property. **Figure** **2**a shows the changes in the sheet resistance of CuNWs electrodes treated with different ionic liquids and exposed in air for 60 days. It can be seen that all of the CuNWs films have been protected to some extent after treatment with the ionic liquids. The CuNWs@[EPy]TFSI composite electrodes present a relatively low oxidation resistance with the sheet resistance increasing from 30.3 to 1018.2 Ω sq^−1^ after exposing in air for 60 days, while the sheet resistance of bare CuNWs electrodes increase from 27.9 to 1735.7 Ω sq^−1^. Interestingly, CuNWs@[EMIM]TFSI, CuNWs@[P2,4,4,4]TFSI and CuNWs@[BMP]TFSI composite electrodes display the comparable oxidation‐resistant property with the sheet resistance increasing from ≈ 30 to 282.0, 348.6, and 459.4 Ω sq^−1^, respectively, wherein the [EMIM]TFSI is among the best of the three kinds of ionic liquids for CuNWs films protection. As expected, [BTAMMIM]TFSI shows the best oxidation‐resistant property for CuNWs films, whose sheet resistance only increases by 9.2 Ω sq^−1^ from 28.2 to 37.4 Ω sq^−1^. Compared to the bare CuNWs, the sheet resistance of the CuNWs@[BTAMMIM]TFSI composite electrodes increases by only 0.54% after 60‐day air exposure. SEM images (Figure 2b) reveal that the CuNWs are severely oxidized for the bare CuNWs electrodes after exposure in air for 60 days while the morphology of the CuNWs@[BTAMMIM]TFSI composite electrode remains unchanged under the same conditions, which is consistent with the conductivity difference between the two electrodes, suggesting the excellent oxidation‐resistant property of the [BTAMMIM]TFSI for CuNWs films.

To clarify the effect of different ionic liquids on the oxidation‐resistant properties of the composite electrodes at the molecular level, QCM measurements are carried out to investigate the molecular interactions between the copper and ionic liquids. As shown in Figure 2c, the frequency changes decrease immediately for all kinds of ionic liquids while injecting them into the Cu‐coated QCM sensors. This suggests that all of the five kinds of ionic liquids can form a stable complex with the copper, which is consistent with the oxidation‐resistant experiments that all kinds of ionic liquids show oxidation‐resistant protection for CuNWs films. However, the frequency changes exhibit obvious difference in five ionic liquids at the same concentration. Taking the ionic liquids concentration of 20 mm as an example, the frequency change for [BTAMMIM]TFSI is 5.37 Hz, while the frequency changes of [EMIM]TFSI, [P2,4,4,4]TFSI, [BMP]TFSI and [EPy]TFSI are 3.86, 3.45, 3.56, and 2.39 Hz, respectively. According to the concentrations and frequency changes, the binding constants (*K*_a_) of [BTAMMIM]TFSI, [EMIM]TFSI, [P2,4,4,4]TFSI, [BMP]TFSI, and [EPy]TFSI with copper (Figure S8, Supporting Information) are calculated to be 17.5, 6.2, 4.3, 3.1, and 2.4 M^−1^, respectively. The results suggest that [BTAMMIM]TFSI can form the most stable complex with copper among the five kinds of ionic liquids, which is consistent with the oxidation‐resistant tests that CuNWs@[BTAMMIM]TFSI composite electrodes show the best oxidation‐resistant property among all composite electrodes.

To further understand the underlying coordination site between the ionic liquids and copper, DFT calculations are carried out using the Vienna Ab initio Simulation Package (VASP).^[^ ^35^ ^]^ As shown in Figure 2d and Figure S9 (Supporting Information), all of the ionic liquids can interact with copper through the coordination effect of the O at the anion of [TFSI]. However, the cations of the ionic liquids have a great influence on the configuration of the ionic liquids, which is then reflected on the coordination interaction between the ionic liquids and copper. Consequently, the adsorption energies (*E*_ads_) for different ionic liquids show obvious difference, which are −6.12, −5.71, −5.49, and −5.41 eV for [EMIM]TFSI, [P2,4,4,4]TFSI, [BMP]TFSI and [EPy]TFSI, respectively. It is worth noting that [BTAMMIM]TFSI can also coordinate with copper through the benzotriazole‐functionalized cations, in addition to the coordination interaction of the anion of [TFSI]. The anion‐cation synergistic coordination effects of the benzotriazole‐functionalized cations and [TFSI] anions afford the [BTAMMIM]TFSI with the highest adsorption energy of −6.90 eV among all of the five kinds of ionic liquids. The calculated results are consistent with the QCM measurements and oxidation‐resistant tests that [BTAMMIM]TFSI has the largest binding constants with copper and exhibits the best oxidation‐resistant property for CuNWs networks. Therefore, the ionic liquid of [BTAMMIM]TFSI is selected to be acted as an antioxidant protective layer for fabrication of high‐stability CuNWs composite electrodes.

### 2.3 Optimization Studies of Superwettability‐Assisted Transfer Strategy for Large‐Area Preparation of CuNWs Composite Electrodes

After acquiring the ionic liquid with excellent oxidation resistance for CuNWs networks, a superwettability‐assisted transfer strategy was combined to attempt to prepare large‐area CuNWs@[BTAMMIM]TFSI composite flexible transparent electrodes. First, the self‐assembly of the CuNWs on air‐water interface was studied by injecting CuNWs dispersion along the vessel wall to form CuNWs films on the water surface. To improve the uniformity of CuNWs films, PVP is used as an additional additive to fine‐tune the self‐assembly of the CuNWs on air‐water interface. As shown in Figure S10 (Supporting Information), when 3% weight of PVP relative to the CuNWs was added to the dispersion, the sheet resistance of the corresponding CuNWs film transferred to a polyethylene terephthalate (PET) substrate shows a standard deviation of 11.9 Ω sq^−1^ after acetic acid treatment for the optimal time of 5 min (Figure S11, Supporting Information) to remove the organics and surface oxides on CuNWs. The large standard deviation indirectly reflects the ununiformity of the CuNWs films. As the proportion of PVP increases, the standard deviation decreases. While increasing the weight ratios of PVP to 9%, the standard deviation of the sheet resistance is as low as 3.2 Ω sq^−1^. Further raising up the weight ratios of PVP (12% and 15%) results in the increase of the sheet resistance of CuNWs films. Therefore, the weight ratio of 9% is the best proportion for PVP to regulate the CuNWs film formation on air–water interface.

**Figure 3:**
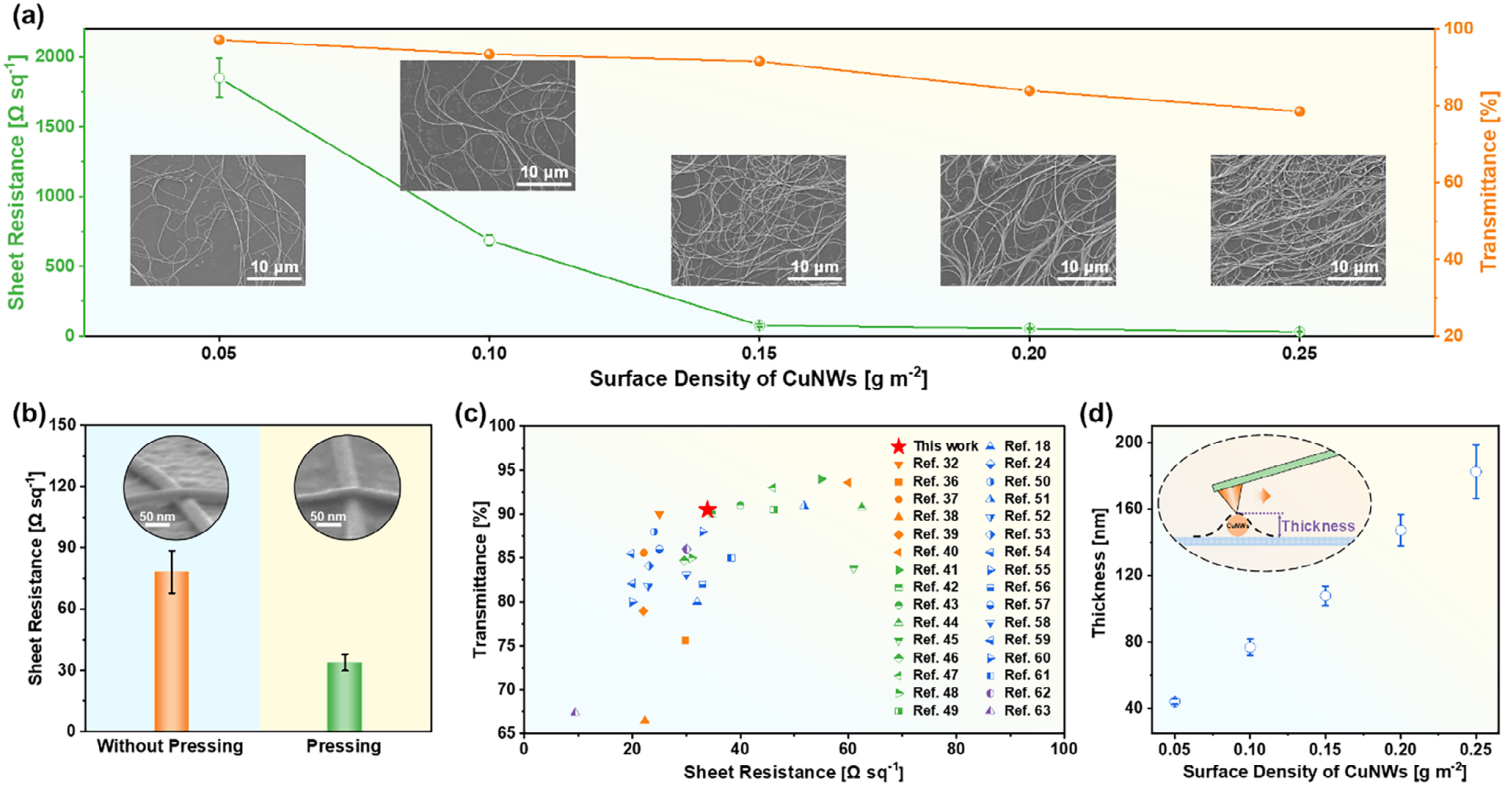
a) Sheet resistance and transmittance of the CuNWs electrodes for different surface densities of CuNWs. The insets show the corresponding SEM images. b) Changes in the sheet resistance and transmittance of the CuNWs electrodes for CuNWs surface density of 0.15 g m^−2^ after pressing with the pressure of 5 MPa. Inset: Tilted SEM images of the CuNWs crosspoint before and after pressure treatment. c) Comparisons of optoelectronic performance for CuNWs electrodes prepared in this work with previously reported CuNWs electrodes constructed by Meyer rod coating^[^ ^32^, ^36^, ^37^, ^38^, ^39^, ^40^ ^]^ (origin), vacuum filtration^[^ ^41^, ^42^, ^43^, ^44^, ^45^, ^46^, ^47^, ^48^, ^49^ ^]^ (green), spray coating^[^ ^18^, ^24^, ^50^, ^51^, ^52^, ^53^, ^54^, ^55^, ^56^, ^57^, ^58^, ^59^, ^60^, ^61^ ^]^(blue), and spin coating^[^ ^62^, ^63^ ^]^ (purple). d) Thickness of the CuNWs electrodes for different surface densities of CuNWs obtained from AFM measurements.

Subsequently, the concentration of CuNWs dispersion injected and the surface density of CuNWs in the films are optimized to acquire the flexible transparent electrodes with high optoelectronic performance. As shown in Figure S12a (Supporting Information), the average sheet resistance of CuNWs films is up to 347.0 Ω sq^−1^, while the concentration of CuNWs injected on the water surface is 0.4 mg mL^−1^. With increasing the concentration of CuNWs to 1.0 mg mL^−1^, the average sheet resistance is decreased as low as 57.7 Ω sq^−1^. Compared with the significant differences of the sheet resistances, the transparency of the CuNWs films prepared with the concentration of CuNWs from 0.4 to 1.0 mg mL^−1^ is only diminished from 93.1% to 90.7% (Figure S12b, Supporting Information). To clarify the great change of the sheet resistances, SEM was used to characterize the morphology of the CuNWs films. It can be seen from Figure S12c (Supporting Information) that the CuNWs are just adjacent to each other and do not form a good cross network while using 0.4 mg mL^−1^ CuNWs dispersion to prepare CuNWs films. When the concentration of CuNWs reaches 1.0 mg mL^−1^, the CuNWs intersect well with each other, thus leading to a high conductivity for the corresponding CuNWs films. In addition, the surface density of CuNWs also has a great effect on the optoelectronic performance of the CuNWs films. As depicted in **Figure** **3**a, with the increase of the surface density of CuNWs, both the sheet resistance and the optical transparency decreased. The sheet resistance of the electrodes is sharply reduced from 1849.6 to 78.2 Ω sq^−1^ with the optical transparency lowered from 97.1% to 91.5% as the surface density of CuNWs increased from 0.05 to 0.15 g m^−2^. When the surface density of CuNWs raised from 0.15 to 0.25 g m^−2^, the sheet resistance is slowly decreased from 78.2 Ω sq^−1^ to 31.2 Ω sq^−1^ but the optical transparency shows significant reduction from 91.5% to 78.5% (Figure S13, Supporting Information). SEM images (Figure 3a) show that CuNWs are not dense enough to support a high sheet resistance of the CuNWs films while the surface density of CuNWs is lower than 0.15 g m^−2^. When the surface density of CuNWs is larger than 0.15 g m^−2^, the CuNWs in the films become dense, thus leading to a low transparency of the corresponding electrodes. The variety of the morphologies of the CuNWs films are consistent with the change trend of their sheet resistance and optical transparency.

To further improve the conductivity of the CuNWs films, mechanical pressure is applied to reducing the junction resistance between two adjacent CuNWs. As shown in Figure 3b and Figure S14 (Supporting Information), the sheet resistance exhibits an apparent decrease after pressing with the optimized pressure of 5 MPa. For example, after pressing for 15 s, the average sheet resistance of the CuNWs films with the surface density of 0.15 g m^−2^ decreases from 78.2 to 33.9 Ω sq^−1^ with a slight reduction of the optical transparency from 91.5% to 90.5% (Figure S15, Supporting Information). This performance is comparable with those performances of the CuNWs films prepared by Meyer rod coating,^[^ ^32^, ^36^, ^37^, ^38^, ^39^, ^40^ ^]^ vacuum filtration,^[^ ^41^, ^42^, ^43^, ^44^, ^45^, ^46^, ^47^, ^48^, ^49^ ^]^ spray coating^[^ ^18^, ^24^, ^50^, ^51^, ^52^, ^53^, ^54^, ^55^, ^56^, ^57^, ^58^, ^59^, ^60^, ^61^ ^]^ and spin coating^[^ ^62^, ^63^ ^]^ (Figure 3c), suggesting the promising prospects of the superwettability‐assisted transfer strategy to prepare large‐area CuNWs electrodes with high optoelectronic performance. Tilted SEM images (Figure 3b) show that the two crossed CuNWs are tightly stacked with each other after treatment with pressure, thus resulting in an enormous decrease of the sheet resistance. In addition, owing to the stacking of CuNWs, the thickness of CuNWs films characterized by Atomic Force Microscope (AFM) is monotonically increased as the increase of the surface density of CuNWs (Figure 3d; Figure S16, Supporting Information).

To achieve the large‐area preparation of air‐stable CuNWs@[BTAMMIM]TFSI composite flexible transparent electrodes, a continuous fabrication processing (Figure 1e), which integrates the superwettability‐assisted transfer strategy and ionic liquid oxidation‐resistant protection, is proposed. By taking advantage of this procedure, a large area of CuNWs@[BTAMMIM]TFSI composite flexible transparent electrodes (Figure 1f) with the size of 40 cm in length and 25 cm in width is successfully fabricated. The composite electrodes show an extremely low sheet resistance of 37.2 Ω sq^−1^ and a high transparency of 88.2% at 550 nm. Importantly, the sheet resistance of the composite electrodes which are divided into 5 × 8 pixels (Figure S17a, Supporting Information) exhibit a uniform distribution with a standard deviation as low as 3.3 Ω sq^−1^ (Figure S17b, Supporting Information). Thanks to the introduction of [BTAMMIM]TFSI which can be clearly characterized by attenuated total reflectance Fourier transform infrared (ATR‐FTIR) spectroscopy (Figure S18, Supporting Information), HRTEM images (Figure S19, Supporting Information) and energy‐dispersive X‐ray spectrometers (EDS) mapping (Figure S20, Supporting Information), the composite electrodes exhibit an excellent oxidation‐resistant behavior than the bare CuNWs electrodes, which is consistent with the aforementioned oxidation‐resistant experiments.

### 2.4 Stability Tests of CuNWs@[BTAMMIM]TFSI Composite Electrode

To evaluate long‐term operational stability, continuous direct current (DC) voltage of 5 V was applied on the CuNWs@[BTAMMIM]TFSI composite electrode to test its ability to resist Joule heating effects. As shown in Figure S21 (Supporting Information), the CuNWs electrode shows an increase of 5.5 times in sheet resistance after electrical loading for 1 h, whereas the sheet resistance of the CuNWs@[BTAMMIM]TFSI composite electrode increased by 0.6 times under the same conditions. In additon to the operational robustness, the CuNWs@[BTAMMIM]TFSI composite electrode can endure the harsh chemical conditions. It can be seen from Figure S22a,b (Supporting Information) that the sheet resistance of CuNWs@[BTAMMIM]TFSI composite electrode immersed in HNO_3_ (pH = 3) and NaOH (pH = 13) solution for 20 min increased by 2.4 times and 1.9 times, respectively, while the sheet resistance of CuNWs electrode show a great increase of 136 times and 133 times, respectively, under the same conditions, indicating the good tolerance of CuNWs@[BTAMMIM]TFSI composite electrode in acidic and basic environments. The ability of resistance to high temperature and high humidity is also important for CuNWs‐based flexible transparent electrode. As shown in Figure S22c (Supporting Information), the CuNWs electrode becomes non‐conductive after placement under the condition of 85 °C and 85% relative humidity (RH) for 1 h. However, the sheet resistance of the CuNWs@[BTAMMIM]TFSI composite electrode only increased by 3.0 times at 85 °C and 85% RH for 2 h, which is attributed to the existence of [BTAMMIM]TFSI that efficiently protects the CuNWs from moisture and oxygen. Furthermore, both CuNWs@[BTAMMIM]TFSI composite electrode and CuNWs electrode show excellent mechanical stability thanks to the intrinsic flexibility of the CuNWs. The bending tests (Figure S23a, Supporting Information) suggest that the sheet resistance of the CuNWs@[BTAMMIM]TFSI composite electrode and CuNWs electrode almost has no significant change with the bending radius changing from 40 to 2.5 mm. And the sheet resistance can keep constant even after 1000 bending cycles at the bending radius of 5.0 mm (Figure S23b, Supporting Information). As a contrast, the ITO films exhibit a sharp increase in sheet resistance during the bending experiments due to the brittleness of ITO. These results demonstrate that the CuNWs@[BTAMMIM]TFSI composite electrode is flexible enough to meet the demand to be applied in the fields of flexible electronics.

### 2.5 Application of CuNWs@[BTAMMIM]TFSI Composite Electrode

**Figure 4:**
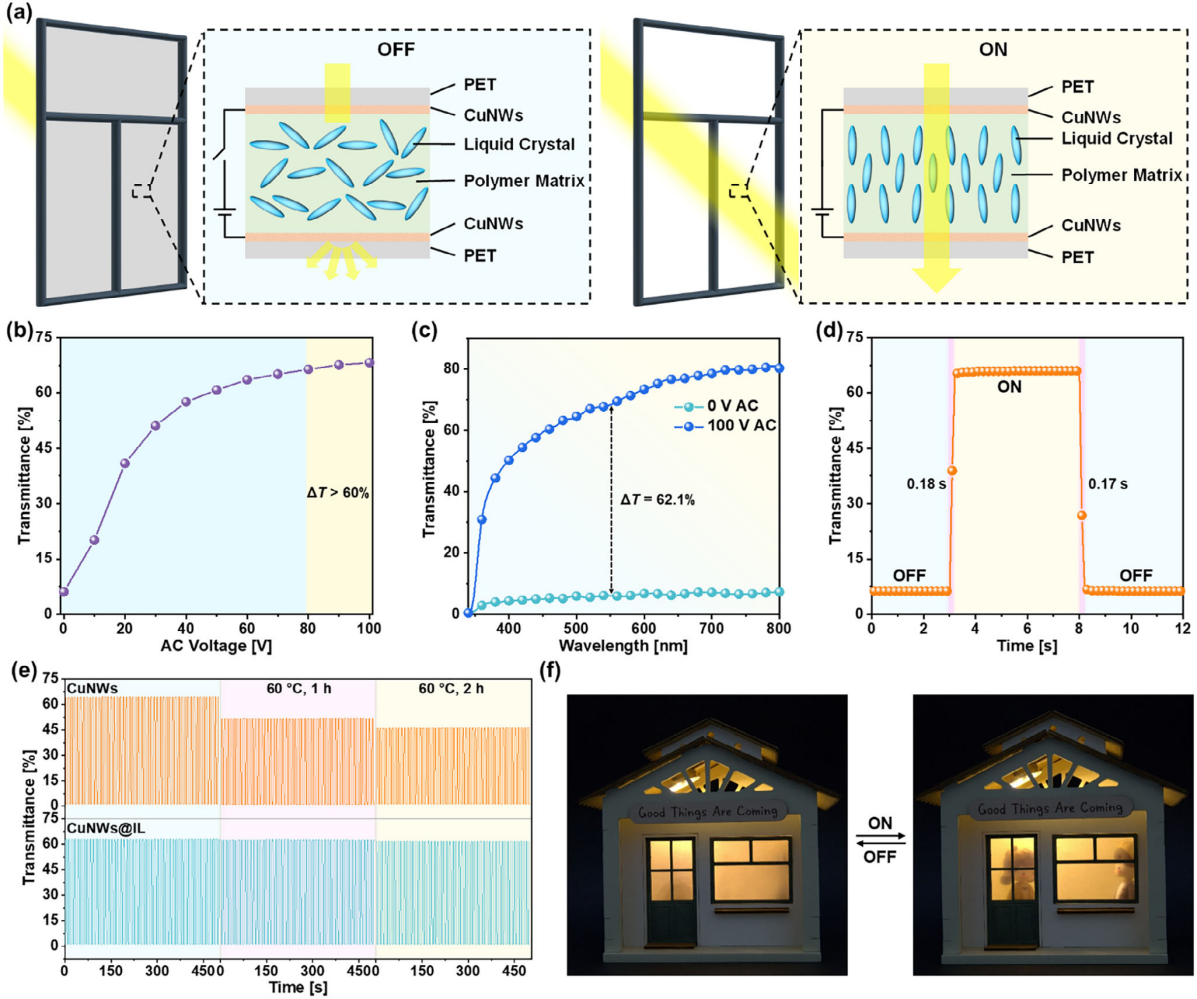
a) Schematic of the application and operation principle of the smart windows fabricated with CuNWs@[BTAMMIM]TFSI composite flexible transparent electrodes. b) Influence of applied voltage between the CuNWs@[BTAMMIM]TFSI composite flexible transparent electrodes on the transmittance of the smart window. c) Transmittance spectra of the smart window in ON and OFF state. d) Response time of the smart windows for ON and OFF state. e) Changes of the transmittance of the smart window fabricated with CuNWs electrodes and CuNWs@[BTAMMIM]TFSI composite flexible transparent electrodes after heating at 60 °C for 1 and 2 h. f) Promising application of the CuNWs@[BTAMMIM]TFSI composite flexible transparent electrodes‐based smart window in intelligent buildings.

To evaluate the practical application of our work, a polymer dispersed liquid crystal (PDLC)‐based smart window is fabricated using the CuNWs@[BTAMMIM]TFSI composite electrode (**Figure** **4**a). When an alternating current (AC) voltage of 100 V is applied between the two composite electrodes, the smart window transitions from opaque (ON state) to transparent (OFF state) with the optical transparency increasing from 6.1% to 68.2% (Figure 4b,c), which is attributed to that the randomly aligned liquid crystals sandwiched between the two composite electrodes become orientationally ordered under the electric field (Figure 4a). Apart from the huge difference in transparency between the ON and OFF state, the respond rate of the smart window is very fast (Figure 4d). The relaxation times are as low as 0.18 s and 0.17 s for the ON and OFF state, respectively. What's more, the transmittance of the smart window fabricated with CuNWs@[BTAMMIM]TFSI composite flexible transparent electrodes is almost unchanged after heating at 60 °C for 2 h, while the smart window fabricated with CuNWs electrodes shows an obvious decrease in transmittance under the same conditions (Figure 4e). This result demonstrates [BTAMMIM]TFSI plays a key role on the good durability of the smart window for long‐term operation. As the model displayed in Figure 4f, Figure S24 and Video S1 (Supporting Information), the excellent performance of the smart window prepared by the CuNWs@[BTAMMIM]TFSI composite electrodes make it succeed in privacy ensurance in intelligent buildings or even curved buildings, which also indicates the prospective applications of the composite electrodes in the flexible optoelectronics fields.

## 3 Conclusion

In summary, we have demonstrated the first introducing the ionic liquid of [BTAMMIM]TFSI as an antioxidant protective layer to prepare air‐stable CuNWs‐based flexible transparent electrodes. The sheet resistance of the CuNWs@[BTAMMIM]TFSI composite flexible transparent electrodes increases by only 0.54% than that of bare CuNWs after 60 days of air exposure. QCM measurements and DFT calculations reveal that the anion‐cation synergistic coordination effects of the benzotriazole‐functionalized cations and [TFSI] anions of the [BTAMMIM]TFSI with copper are the key factors to contribute to the high oxidation‐resistant performance of the composite electrodes. Through integrating with the superwettability‐assisted transfer strategy, large‐area composite electrodes (40 × 25 cm^2^) with a low sheet resistance of 37.2 Ω sq^−1^ and a high transparency of 88.2% at 550 nm are successfully fabricated, which also show good tolerance to the harsh conditions, such as acidic, basic, high‐temperature and high‐humidity environments. A smart window fabricated with the CuNWs@[BTAMMIM]TFSI composite flexible transparent electrodes displays excellent performance of high transmittance variation, fast response and good durability, demonstrating the promising applications of the CuNWs@[BTAMMIM]TFSI composite electrodes in the field of flexible optoelectronics. Further studies on the antioxidant protection of the CuNWs‐based flexible transparent electrodes are still ongoing in our laboratory.

## Conflict of Interest

The authors declare no conflict of interest.

## Supporting information



Supporting Information

Supplemental Video 1

## Data Availability

The data that support the findings of this study are available from the corresponding author upon reasonable request.

## References

[1] Y. Zhang , S.‐W. Ng , X. Lu , Z. Zheng , Chem. Rev. 2020, 120, 2049. 31961135 10.1021/acs.chemrev.9b00483

[2] S. Li , Z. Li , X. Wan , Y. Chen , eScience 2023, 3, 100085.

[3] S. Chang , J. H. Koo , J. Yoo , M. S. Kim , M. K. Choi , D.‐H. Kim , Y. M. Song , Chem. Rev. 2024, 124, 768. 38241488 10.1021/acs.chemrev.3c00548

[4] S. Yuan , Z. Fan , G. Wang , Z. Chai , T. Wang , D. Zhao , A. A. Busnaina , X. Lu , Adv. Sci. 2023, 10, 2304990. 10.1002/advs.202304990 PMC10700185 37818769

[5] H.‐C. Chu , Y.‐C. Chang , Y. Lin , S.‐H. Chang , W.‐C. Chang , G.‐A. Li , H.‐Y. Tuan , ACS Appl. Mater. Interfaces 2016, 8, 13009. 27144911 10.1021/acsami.6b02652

[6] D. Won , J. Bang , S. H. Choi , K. R. Pyun , S. Jeong , Y. Lee , S. H. Ko , Chem. Rev. 2023, 123, 9982. 37542724 10.1021/acs.chemrev.3c00139 PMC10452793

[7] J. Song , H. Zeng , Angew. Chem., Int. Ed. 2015, 54, 9760. 10.1002/anie.201501233 26223702

[8] L. Yu , C. Shearer , J. Shapter , Chem. Rev. 2016, 116, 13413. 27704787 10.1021/acs.chemrev.6b00179

[9] S. Pang , Y. Hernandez , X. Feng , K. Müllen , Adv. Mater. 2011, 23, 2779. 21520463 10.1002/adma.201100304

[10] X. Lu , Y. Zhang , Z. Zheng , Adv. Electron. Mater. 2021, 7, 2001121.

[11] S. Ye , A. R. Rathmell , Z. Chen , I. E. Stewart , B. J. Wiley , Adv. Mater. 2014, 26, 6670. 25252266 10.1002/adma.201402710

[12] J. Ouyang , SmartMat 2021, 2, 263.

[13] J. Yang , L. Chang , H. Zhao , X. Zhang , Z. Cao , L. Jiang , InfoMat 2024, 6, 12529.

[14] I. E. Stewart , S. Ye , Z. Chen , P. F. Flowers , B. J. Wiley , Chem. Mater. 2015, 27, 7788.

[15] U. S. Geological Survey, “Mineral Commodity Summaries ”, 2024.

[16] L. Zhao , J. Li , Z. Song , X. Wang , S. Yu , ACS Appl. Nano Mater. 2023, 6, 10658.

[17] H. Dong , C. Chang , Q. Tan , L. Zhao , P. Yang , S. Yu , ACS Appl. Nano Mater. 2023, 6, 10384.

[18] B. Zhang , W. Li , J. Jiu , Y. Yang , J. Jing , K. Suganuma , C.‐F. Li , Inorg. Chem. 2019, 58, 3374. 30789711 10.1021/acs.inorgchem.8b03460

[19] Y. Zhu , M. C. Hartel , N. Yu , P. R. Garrido , S. Kim , J. Lee , P. Bandaru , S. Guan , H. Lin , S. Emaminejad , N. R. de Barros , S. Ahadian , H.‐J. Kim , W. Sun , V. Jucaud , M. R. Dokmeci , P. S. Weiss , R. Yan , A. Khademhosseini , Small Methods 2022, 6, 2100900. 10.1002/smtd.202100900 PMC8852346 35041280

[20] L. Dou , F. Cui , Y. Yu , G. Khanarian , S. W. Eaton , Q. Yang , J. Resasco , C. Schildknecht , K. Schierle‐Arndt , P. Yang , ACS Nano 2016, 10, 2600. 26820809 10.1021/acsnano.5b07651

[21] W. Gaynor , G. F. Burkhard , M. D. McGehee , P. Peumans , Adv. Mater. 2011, 23, 2905. 21538594 10.1002/adma.201100566

[22] W. Gaynor , J.‐Y. Lee , P. Peumans , ACS Nano 2010, 4, 30. 20025290 10.1021/nn900758e

[23] L. Shi , R. Wang , H. Zhai , Y. Liu , L. Gao , J. Sun , Phys. Chem. Chem. Phys. 2015, 17, 4231. 25571983 10.1039/c4cp05187d

[24] S. Polat Genlik , D. Tigan , Y. Kocak , K. E. Ercan , M. O. Cicek , S. Tunca , S. Koylan , S. Coskun , E. Ozensoy , H. E. Unalan , ACS Appl. Mater. Interfaces 2020, 12, 45136. 32896125 10.1021/acsami.0c11729

[25] T. Zhang , M. Zhao , F. Daneshvar , F. Xia , H.‐J. Sue , ACS Appl. Nano Mater. 2019, 2, 7775.

[26] I. E. Stewart , PhD Dissertation Thesis, Duke University, Durham North Carolina 2016.

[27] J. Peng , B. Chen , Z. Wang , J. Guo , B. Wu , S. Hao , Q. Zhang , L. Gu , Q. Zhou , Z. Liu , S. Hong , S. You , A. Fu , Z. Shi , H. Xie , D. Cao , C.‐J. Lin , G. Fu , L.‐S. Zheng , Y. Jiang , N. Zheng , Nature 2020, 586, 390. 33057223 10.1038/s41586-020-2783-x

[28] L. Chang , X. Zhang , Y. Ding , H. Liu , M. Liu , L. Jiang , ACS Appl. Mater. Interfaces 2018, 10, 29010. 30080390 10.1021/acsami.8b09023

[29] J. Wang , C. Teng , Y. Jiang , Y. Zhu , L. Jiang , Adv. Mater. 2019, 31, 1806742. 10.1002/adma.201806742 30633824

[30] C. Ma , X. Chen , Y. Zhang , C. Liu , H. Liu , S. Diao , ACS Appl. Nano Mater. 2024, 7, 25078.

[31] X. Chen , Y. Zhang , C. Ma , H. Liu , Chem. Eng. J. 2023, 476, 146505.

[32] T.‐H. Duong , H.‐C. Kim , Ind. Eng. Chem. Res. 2018, 57, 3076.

[33] C. Kang , S. Yang , M. Tan , C. Wei , Q. Liu , J. Fang , G. Liu , ACS Appl. Nano Mater. 2018, 1, 3155.

[34] J. P. Cason , M. E. Miller , J. B. Thompson , C. B. Roberts , J. Phys. Chem. B 2001, 105, 2297.

[35] G. Kresse , J. Hafner , Phys. Rev. B 1993, 47, 558. 10.1103/physrevb.47.558 10004490

[36] J. Kim , J. W. Lim , F. M. Mota , J.‐E. Lee , R. Boppella , K. Y. Lim , K. Kim , W. K. Choi , D. H. Kim , Nanoscale 2016, 8, 18938. 27740663 10.1039/c6nr05460a

[37] Z. Zhong , K. Woo , I. Kim , H. Hwang , S. Kwon , Y.‐M. Choi , Y. Lee , T.‐M. Lee , K. Kim , J. Moon , Nanoscale 2016, 8, 8995. 27074548 10.1039/c6nr00444j

[38] X. Zhang , Z. Tang , D. Tian , K. Liu , W. Wu , Mater. Res. Bull. 2017, 90, 175.

[39] L. Lian , D. Dong , H. Wang , G. He , Org. Electron. 2019, 65, 70.

[40] N.‐H. Tran , H.‐M. Hoang , T.‐H. Duong , H.‐C. Kim , Appl. Surf. Sci. 2020, 520, 146216.

[41] C. Mayousse , C. Celle , A. Carella , J.‐P. Simonato , Nano Res. 2014, 7, 315.

[42] F. Cui , Y. Yu , L. Dou , J. Sun , Q. Yang , C. Schildknecht , K. Schierle‐Arndt , P. Yang , Nano Lett. 2015, 15, 7610. 26496181 10.1021/acs.nanolett.5b03422

[43] D. Kim , J. Kwon , J. Jung , K. Kim , H. Lee , J. Yeo , S. Hong , S. Han , S. H. Ko , Small Methods 2018, 2, 1800077.

[44] Y. Wang , P. Liu , B. Zeng , L. Liu , J. Yang , Nanoscale Res. Lett. 2018, 13, 78. 29516262 10.1186/s11671-018-2486-5 PMC5842171

[45] H. Xiang , T. Guo , M. Xu , H. Lu , S. Liu , G. Yu , ACS Appl. Nano Mater. 2018, 1, 3754.

[46] P. Liu , J. Wang , J. I. E. Cheng , L. Liu , H. Wang , B. Zeng , F. Chi , Surf. Rev. Lett. 2019, 27, 1950171.

[47] S.‐S. Chee , H. Kim , M. Son , M.‐H. Ham , Electron. Mater. Lett. 2020, 16, 404.

[48] H. Zhang , S. Wang , Y. Tian , J. Wen , C. Hang , Z. Zheng , Y. Huang , S. Ding , C. Wang , Nano Mater. Sci. 2020, 2, 164.

[49] Y. Zhao , X. Zhou , W. Huang , J. Kang , G. He , Org. Electron. 2023, 113, 106690.

[50] C. Sachse , N. Weiß , N. Gaponik , L. Müller‐Meskamp , A. Eychmüller , K. Leo , Adv. Energy Mater. 2014, 4, 1300737.

[51] Y. Ahn , Y. Jeong , D. Lee , Y. Lee , ACS Nano 2015, 9, 3125. 25712446 10.1021/acsnano.5b00053

[52] S. Ding , J. Jiu , Y. Tian , T. Sugahara , S. Nagao , K. Suganuma , Phys. Chem. Chem. Phys. 2015, 17, 31110. 26536570 10.1039/c5cp04582g

[53] C. Hwang , J. An , B. D. Choi , K. Kim , S.‐W. Jung , K.‐J. Baeg , M.‐G. Kim , K. M. Ok , J. Hong , J. Mater. Chem. C 2016, 4, 1441.

[54] H. Hwang , A. Kim , Z. Zhong , H.‐C. Kwon , S. Jeong , J. Moon , Adv. Funct. Mater. 2016, 26, 6545.

[55] C. Celle , A. Cabos , T. Fontecave , B. Laguitton , A. Benayad , L. Guettaz , N. Pélissier , V. H. Nguyen , D. Bellet , D. Muñoz‐Rojas , J.‐P. Simonato , Nanotechnology 2018, 29, 085701. 29339582 10.1088/1361-6528/aaa48e

[56] S. Ding , Y. Tian , J. Jiu , K. Suganuma , RSC Adv. 2018, 8, 2109. 35542590 10.1039/c7ra12738c PMC9077247

[57] D. Tigan , S. P. Genlik , B. Imer , H. E. Unalan , Nanotechnology 2019, 30, 325202. 30991365 10.1088/1361-6528/ab19c6

[58] L. Zhao , S. Yu , X. Li , M. Wu , L. Li , Sol. Energy Mater. Sol. Cells 2019, 201, 110067.

[59] Y. Sun , C. Du , M. Wu , L. Zhao , S. Yu , B. Gong , Q. Ding , Nanotechnology 2020, 31, 375303. 32454475 10.1088/1361-6528/ab967b

[60] S. Yu , Z. Liu , L. Zhao , B. Gong , Opt. Mater. 2021, 119, 111301.

[61] J.‐M. Chiu , I. Wahdini , Y.‐N. Shen , C.‐Y. Tseng , J. Sharma , Y. Tai , ACS Appl. Energy Mater. 2023, 6, 5058.

[62] D. V. R. Kumar , A. M. Koshy , N. Sharma , N. Thomas , P. Swaminathan , ACS Omega 2023, 8, 21107. 37332811 10.1021/acsomega.3c02048 PMC10269267

[63] S. Li , Y. Chen , L. Huang , D. Pan , Inorg. Chem. 2014, 53, 4440. 24750021 10.1021/ic500094b

