# Supplementary material for: Benzotriazole‐Functionalized Ionic Liquid and Superwettability‐Assisted Transfer Enable Air‐Stable, Large‐Area Copper Nanowires‐Based Flexible Transparent Electrodes
Source: Adv Sci (Weinh). 2025 Nov 3;13(4):e15330. doi: 10.1002/advs.202515330 (PMC12822452; doi:10.1002/advs.202515330)
Supplement: Supplementary file 1 — Supporting Information [file ADVS-13-e15330-s001.pdf]

Benzotriazole-Functionalized Ionic Liquid and Superwettability-Assisted Transfer Enable Air-Stable, Large-Area Copper Nanowires-Based Flexible Transparent Electrodes

Bin Hou,[a] Kaiyan Wu,[a] Yuying Deng,[a] Shuo Wang,[a] Wei Wang,[a] Hongqin Wang,[a] Dong Ding,[a] Chuao Ma,*[b] Honglei Fan,[a,b] Hongliang Liu,*[a,b] and Lei Jiang[a,c]

[a] Shandong Laboratory of Advanced Materials and Green Manufacturing at Yantai, Yantai 264006, China

[b] School of Chemistry and Chemical Engineering, Yantai University, Yantai 264005, China

[c] Key Laboratory of Bio-inspired Materials and Interfacial Science, Technical Institute of Physics and Chemistry, Chinese Academy of Sciences, Beijing 100190, China

*E-mail: liuhongliang@ytu.edu.cn (Hongliang Liu)

machuao@ytu.edu.cn (Chuao Ma)

1. Materials

Copper(II) chloride dihydrate, 1-hexadecylamine (HDA), glucose, isopropanol, *n*-hexane, 1-methylimidazole, lithium bis(trifluoromethanesulphonyl)imide (LiTFSI), acetic acid and polyvinyl pyrrolidone (PVP, Mw: 10,000) were purchased from Shanghai Macklin Biochemical Technology Co., Ltd. (China). Dichloromethane and ethanol were purchased from Sinopharm Chemical Reagent Beijing Co., Ltd. (China). 1-(Chloromethyl)-1H-benzotriazole was purchased from Shanghai Titan Scientific Co., Ltd. (China). Polymer dispersed liquid crystals (PDLC) was purchased from Qingdao Qy Liquid Crystal Co., Ltd. (China). Poly(styrene-co-divinylbenzene) was purchased from Merck KGaA (Darmstadt, Germany). Polyethylene terephthalate (PET, ca. 100 μm thickness) was purchased from Yantai Bona Experimental Equipment Co., Ltd. (China). All commercially available chemicals are of analytical grade and were used as received without further purification.

2. Synthesis of copper nanowires (CuNWs)

CuNWs with a length of 100 ± 28 μm and a diameter of 48 ± 12 nm were prepared according to the reported method.[1,2] HDA (3 g, 12.4 mmol, 4.4 equiv.) was added to a solution of $CuCl_2{\cdot}2H_2O$ (0.48 g, 2.82 mmol, 1 equiv.), glucose (0.204 g, 1.13 mmol, 0.4 equiv.) in 160 mL deionized water. After stirring at 60 °C for 2 h, the reaction mixture was poured into a Teflon-lined stainless steel autoclave of 200 mL capacity and heated in an oven at 140 °C for 14 h. After cooling down to room temperature, the products were obtained by centrifugation of 6000 rpm for 5 min. Subsequently, the precipitates were dispersed in deionized water and *n*-hexane was added to the aqueous suspension to separate CuNWs and copper nanoparticles. After vigorously vortexing for 30 s, the CuNWs were extracted in the top *n*-hexane phase. The *n*-hexane phase was then transferred to a 100 mL centrifugal tube. The CuNWs were centrifuged and washed with dichloromethane and isopropanol for three times, respectively. Finally, the CuNWs were dispersed in isopropanol for further use.

3. Preparation of CuNWs Films

The CuNWs suspension (1 mg mL^{-1} in isopropanol containing 0.09 mg mL^{-1} PVP) was injected into the water along the vessel wall with the injection speed of 120 mL h^{-1} and the CuNWs thin film formed at gas/water interface can be observed. Then, a wetted PET substrate, which has been treated with vacuum plasma cleaner for 90 s, was inserted into the gas/water interface. Under the driving force of the surface tension difference between the dispersive solvent and water, CuNWs thin film was transfered and deposited on the PET substrate. Subsequently, the CuNWs/PET film was immersed in glacial acetic acid for 5-8 min and washed with ethanol to remove the residual acid. After drying in air at room temperature, the CuNWs/PET film was pressed by using a tablet press with 5 MPa pressure for 15 s to improve the conductivity of the film.

4. Synthesis of Ionic Liquids

1-((1H-benzo[*d*][1,2,3]triazol-1-yl)methyl)-3-methylimidazoliumbis((trifluoromethyl)sulfonyl)-imide ([BTAMMIM]TFSI) was synthesized according to the reported literature[3]. 1-Methylimidazole (1.2 mL, 15 mmol) was added to a solution of 1-(chloromethyl)-1H-benzo[*d*][1,2,3]triazole (2.5 g, 15 mmol) in 20 mL isopropanol. Then, the reaction was heated at 80 °C for 36 h. After cooling down to room temperature, the reaction mixture was concentrated under reduced pressure. The residue was purified by recrystallization (CH_2Cl_2/PE) to afford the [BTAMMIM]Cl (3.0 g, 80%). A 15 mL aqueous solution containing LiTFSI (2.8 g, 9.6 mmol) was slowly added to a 20 mL aqueous solution of [BTAMMIM]Cl (2.0 g, 8.0 mmol). The anion-exchange reaction was stirred at room temperature for 24 h. Then, the reaction mixture was extracted with CH_2Cl_2. The organic layer was concentrated under reduced pressure. The residue was purified by recrystallization (CH_2Cl_2) to afford the [BTAMMIM]TFSI (3.3 g, 83%). ^{1}H NMR (400 MHz, Acetone-d_6) δ: 9.51 (s, 1H), 8.11 (d, J = 9.3 Hz, 2H), 8.08 (t, J = 1.9 Hz, 1H), 7.81 (t, J = 1.8 Hz, 1H), 7.73-7.66 (m, 1H), 7.56-7.50 (m, 1H), 7.43 (s, 2H), 4.11 (s, 3H); ^{19}F NMR (376 MHz, Acetone-d_6) δ: -79.86.

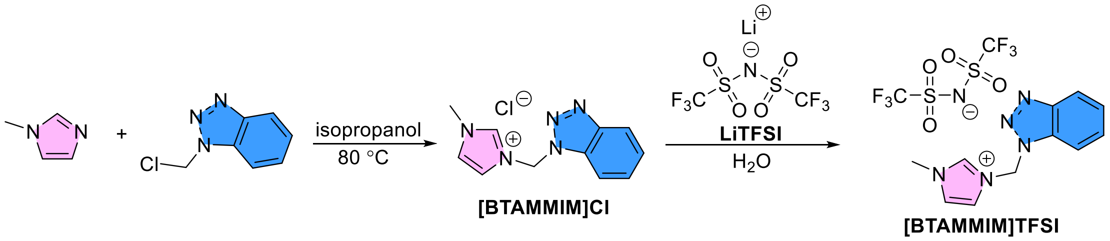

Scheme S1. Synthetic route to [BTAMMIM]TFSI.

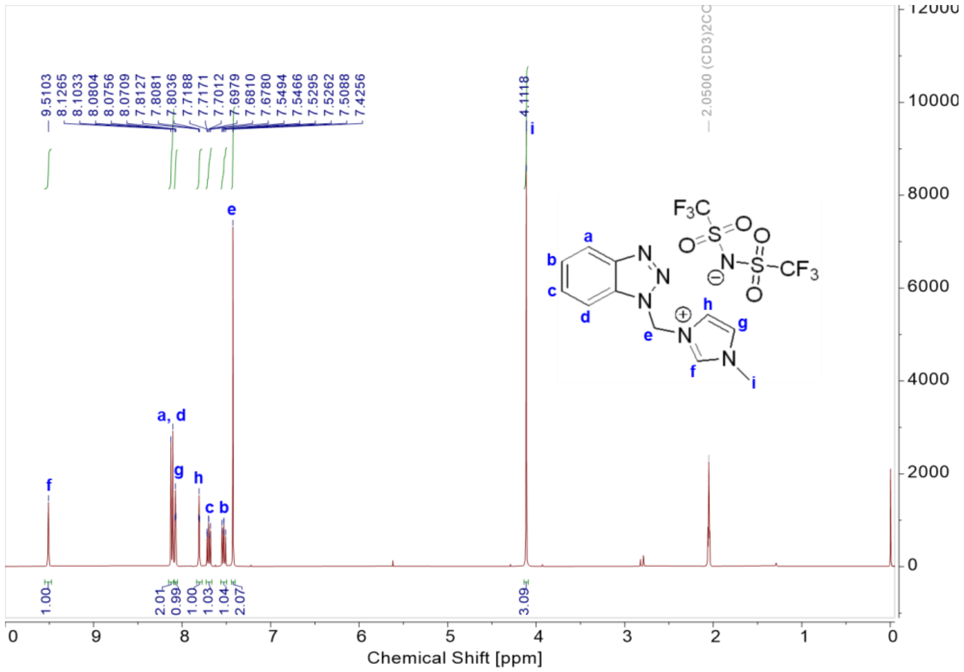

Figure S1. ^{1}H NMR spectrum (400 MHz, Acetone-d_6) of [BTAMMIM]TFSI.

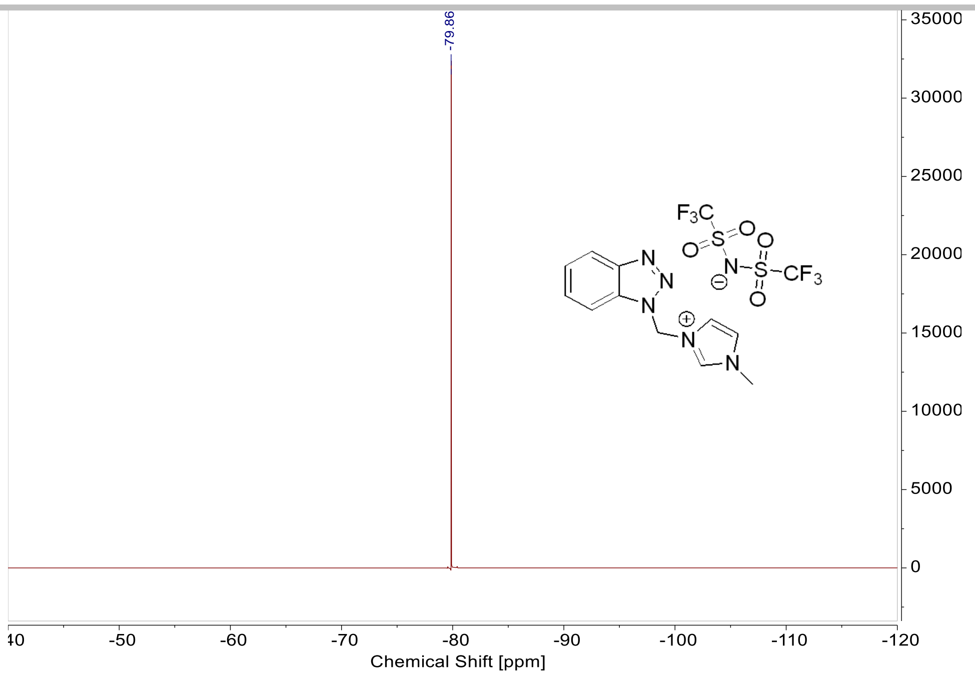

Figure S2. ^{19}F NMR spectrum (376 MHz, Acetone-d_6) of [BTAMMIM]TFSI.

5. Encapsulation of CuNWs Films by Ionic Liquids

Firstly, [BTAMMIM]TFSI (100 mg) was dissolved in 10 mL ethanol. Then, the CuNWs/PET film was immersed in the [BTAMMIM]TFSI solution for 10 s, after which a thin [BTAMMIM]TFSI shell was coated on the surface of the CuNWs. Finally, the CuNWs/PET film was dried in air at room temperature.

6. Fabrication of smart window

The smart window was constructed by sandwiching a PDLC film between two layers of CuNWs electrodes. Firstly, poly(styrene-*co*-divinylbenzene) (10 mg) was added to a solution of PDLC (5 g) and stirred thoroughly to form a homogeneous solution. Then, the mixed PDLC solution was injected into the space between two CuNWs electrodes. Finally, the smart window was obtained by photo-polymerizing the CuNWs electrode/PDLC/CuNWs electrode for 15 min.

7. Surface Tension Measurements

The measurements of surface tension were based on pendant drop method. When the drop is large enough, its shape significantly differs from a spherical shape due to its own mass, whose geometric representation is shown in Figure S3.

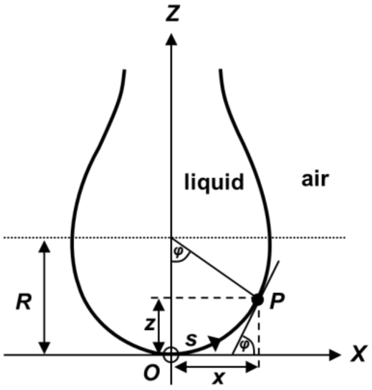

Figure S3. Schematic diagram of a pendant drop profile

The shape of the pendant drop is mainly due to the gravitation and surface tension. This force equilibrium is mathematically exactly defined with the Young-Laplace equation (eq 1)[4].

$$\frac{\partial\varphi}{\partial s} = \frac{2}{R} - \frac{(\rho_{\text{liquid}} - \rho_{\text{air}})gz}{\gamma} - \frac{\sin\varphi}{x} \qquad (1)$$

where φ is the angle with respect to a tangent on point *P*, *s* is the arc length along the drop shape between the point *O* and point *P*, *R* is the radius of curvature relative to the apex, ρ_{liquid} and ρ_{air} are the densities of the samples and the surrounding air phase, *g* is the gravitational acceleration of 9.81 m s^{-2}, *z* is the height of the point *P* from the horizontal axis, γ is the surface tension, *x* is the distance of the point *P* from the vertical axis.

According to the shape and the size of the corresponding pendant drop (Figure S4), the surface tensions of dispersive solvent (isopropanol containing 0.09 mg mL^{-1} PVP) and water were calculated to be 20.9 and 72.2 mN m^{-1}, respectively.

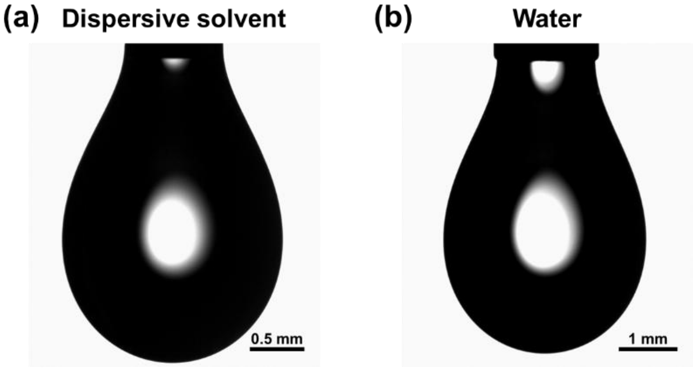

Figure S4. Pendant drops of (a) dispersive solvent and (b) water.

8. QCM Measurements

The QCM measurements were carried out at room temperature with a Q-Sense E1 system (Sweden). Firstly, the QCM channel was washed with ethanol before binding tests of ionic liquids and copper-coated QCM sensors. Then, the ionic liquids diluted with ethanol were

injected into the channel with a flow rate of 100 μL min^{-1}. Finally, the binding curves were obtained by a Q-Sense software and analyzed by QTools.

9. DFT calculations

The DFT calculations were conducted using the Vienna Ab initio Simulation Package (VASP). The exchange-correlation potential was calculated by the Perdew−Burke−Ernzerhof (PBE) generalized gradient approach. The electron-ion interactions and van der Waals (vdW) interactions were studied by the projector augmented wave (PAW) and DFT-D3 method, respectively. All DFT calculations were performed with a cut-off energy of 400 eV, and the 2×2×1 Gamma centered Monkhorst-Pack grids k-points were chosen to sample the Brillouin zone integration. The energy and force convergence criteria of the self-consistent iteration were carried out to be 10^{-4} eV and 0.05 eV $Å^{-1}$, respectively.

10. Characterization

The X-ray powder diffraction (XRD) patterns were measured by a Rigaku SmartLab X-ray Diffractometer. The scanning electron microscopy (SEM) images were characterized on a JEOL JSM-7900F Schottky Field Emission Scanning Electron Microscope operating at 2.0 kV. The high-resolution transmission electron microscopy (HRTEM) images were obtained on a Talos F200X G2 Transmission Electron Microscope working at 200 kV. The surface tensions were measured on a Dataphysics OCA 25 instrument and the needles diameters were 0.91 and 1.8 mm for dispersive solvent and water, respectively. The atomic force microscopy (AFM) images were characterized on a Bruker Dimension Icon Atomic Force Microscope. The attenuated total reflectance Fourier transform infrared (ATR-FTIR) spectra were measured on a Shimadzu IRTracer-100 Fourier Transform Infrared Spectrophotometer. The optical transmittance spectra were conducted by a Shimadzu UV-1900i UV-VIS Spectrophotometer and corrected with a clear PET substrate. The sheet resistances were carried out on a 4Probes Tech ST-21 Four-Point Probe Sheet Resistance Measurement System. The hydrophilic treatment of PET substrates was conducted by a Sunjune VP-RS Series 20 L Vacuum Plasma Cleaner. The bending tests were carried out on a Thorlabs LTS150/M Translation Stage with Stepper Motor. The constant temperature and humidity conditions were provided by a Lugong ST-80LA Humidity Chamber.

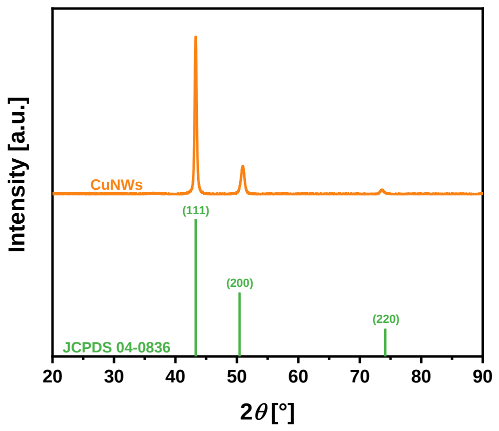

Figure S5. XRD pattern of as-synthesized CuNWs after purification. The diffraction peaks can be matched well to the cubic phase of Cu (JCPDS #04−0836).

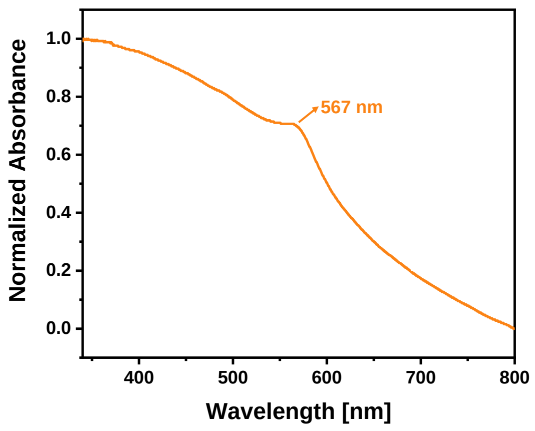

Figure S6. UV-visible absorption spectrum of as-synthesized CuNWs dispersed in isopropanol.

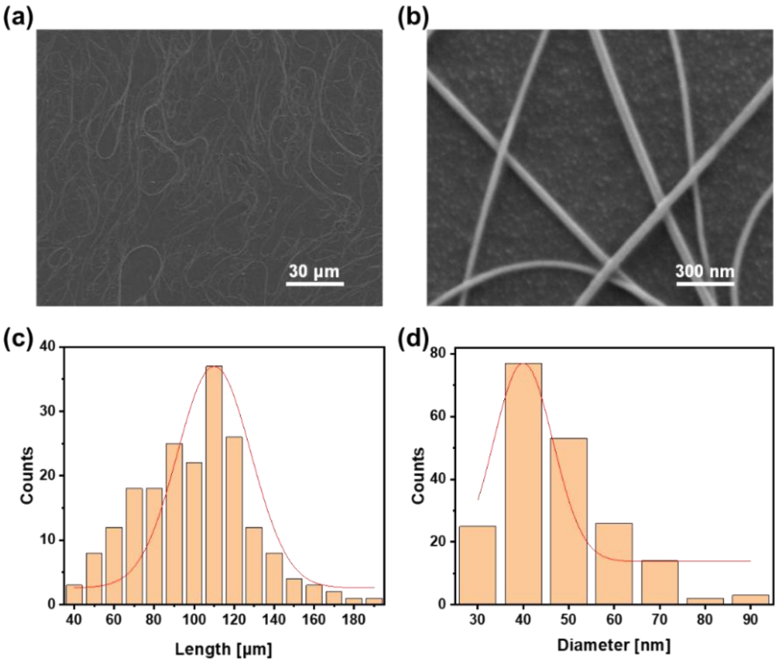

Figure S7. (a, b) SEM images of as-synthesized CuNWs after purification. (c) Length and (d) diameter distributions of 200 randomly selected CuNWs. The average length and diameter are 100 μm and 48 nm, respectively, corresponding to an aspect ratio (length/diameter) of over 2000.

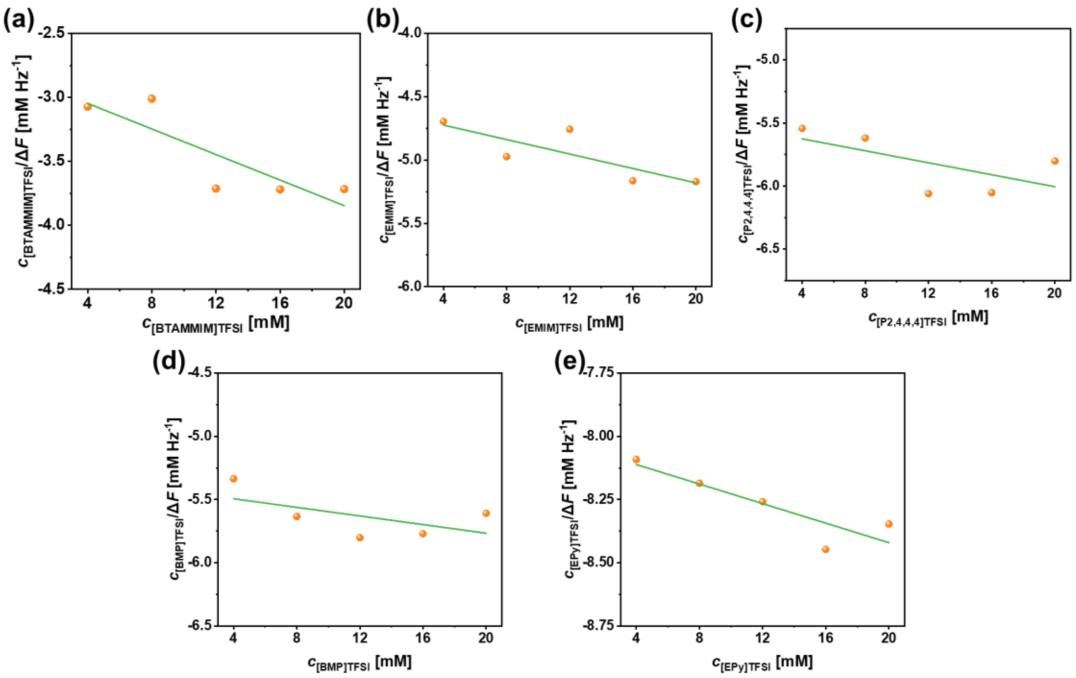

Figure S8. Linear reciprocal plots of (a) $c_{[BTAMMIM]TFSI}/\Delta F$ vs $c_{[BTAMMIM]TFSI}$, (b) $c_{[EMIM]TFSI}/\Delta F$ vs $c_{[EMIM]TFSI}$, (c) $c_{[P,2,4,4]TFSI}/\Delta F$ vs $c_{[P,2,4,4]]TFSI}$, (d) $c_{[BMP]TFSI}/\Delta F$ vs $c_{[BMP]TFSI}$ and (e) $c_{[EPy]TFSI}/\Delta F$ vs $c_{[EPy]TFSI}$ with the corresponding binding constant (K_a) for copper and different ionic liquid systems obtained from QCM measurement.

Based on the frequency changes at the different concentrations of ionic liquids recorded by QCM measurements, the binding constant (K_a) for the ionic liquids and copper systems can be evaluated by eq 2[5,6]. According to the well-known Sauerbrey equation (eq 3)[7], the eq 2 can be recast as eq 4.

$$\frac{c_{\mathrm{IL}}}{\Delta m} = \frac{c_{\mathrm{IL}}}{\Delta m_{\max}} + \frac{1}{\Delta m_{\max} K_a} \qquad (2)$$

$$\Delta f = -\frac{2 f_0^2 \Delta m}{\mathrm{A}\sqrt{\rho_q \mu_q}} \qquad (3)$$

$$\frac{c_{\mathrm{IL}}}{\Delta f} = \frac{c_{\mathrm{IL}}}{\Delta f_{\max}} + \frac{1}{\Delta f_{\max} K_a} \qquad (4)$$

where K_a is the binding constant for ionic liquids and copper systems, $\Delta m_{\max}$ is the maximum binding amount, Δm is the measured binding amount, and c_{IL} is the original concentration of ionic liquids, Δf is the measured frequency shift, f_0 is the frequency of the quartz crystal prior to a mass change, A is the piezoelectrically active area, ρ_q is the density of quartz, and μ_q is the shear modulus, $\Delta f_{\max}$ is the maximum frequency shift.

Figure S8 shows the plot of $c_{\mathrm{[BTAMMIM]TFSI}}$/ ΔF versus $c_{\mathrm{[BTAMMIM]TFSI}}$ based on the data of QCM measurements (Figure 2c). According to the eq 4, the K_a for the different ionic liquids and copper systems can be obtained from the ratio of the slope to the intercept in Figure S8.

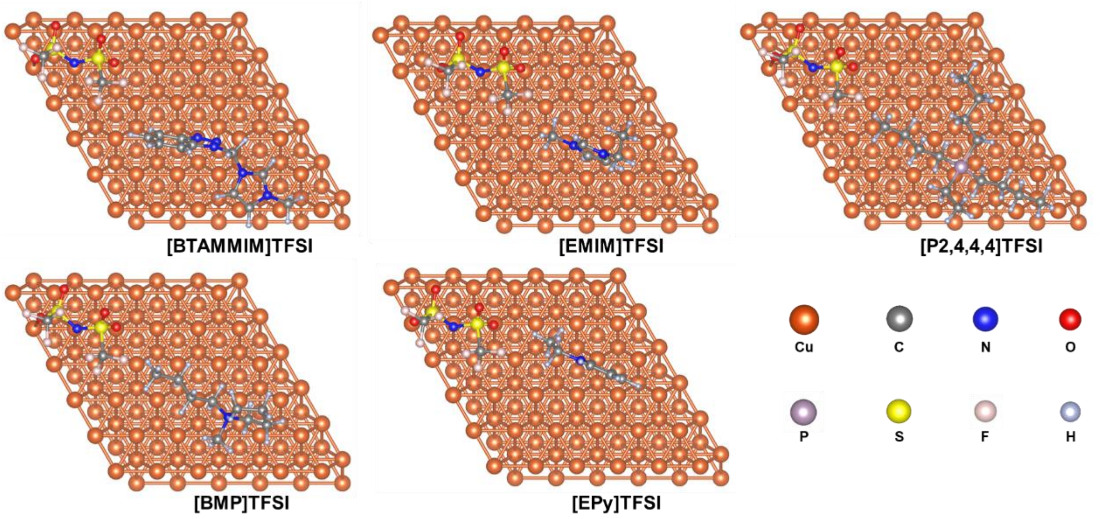

Figure S9. Top views of optimized structures and coordination sites of different ionic liquids on copper obtained by DFT calculations.

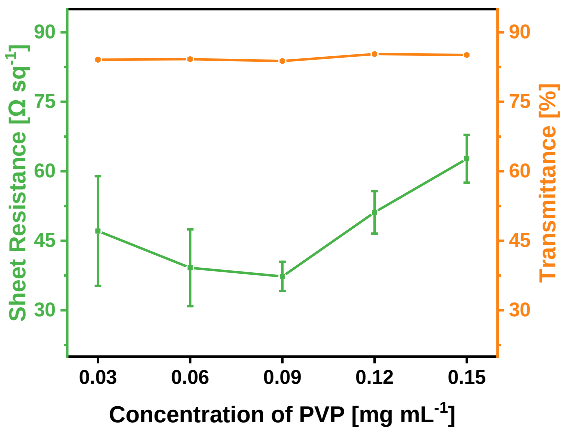

Figure S10. Effect of concentration of PVP on the sheet resistance and transmittance of the CuNWs electrodes.

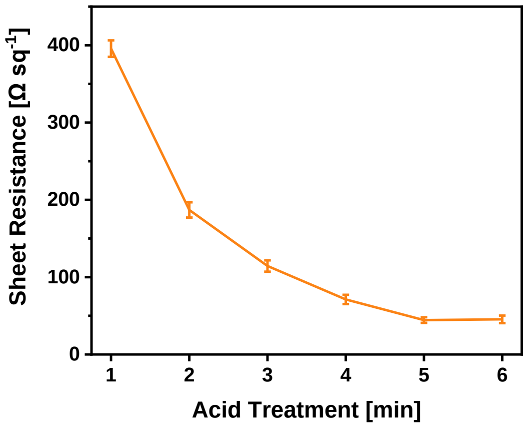

Figure S11. Influence of acid treatment time on the sheet resistance of the CuNWs electrodes.

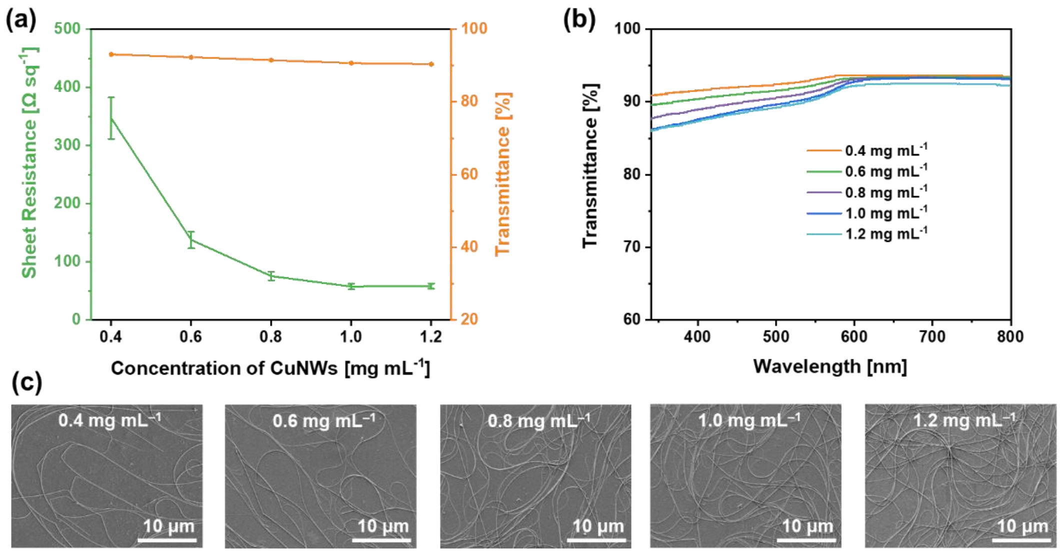

Figure S12. (a) Sheet resistance and transmittance, (b) transmittance spectra and (c) SEM images of the CuNWs electrodes prepared by different concentration of CuNWs.

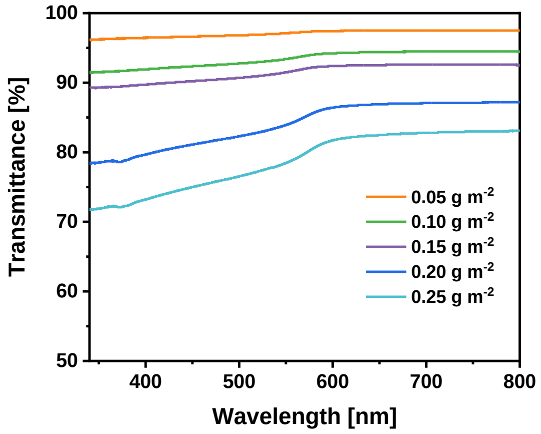

Figure S13. Transmittance spectra of the CuNWs electrodes for different surface densities of CuNWs.

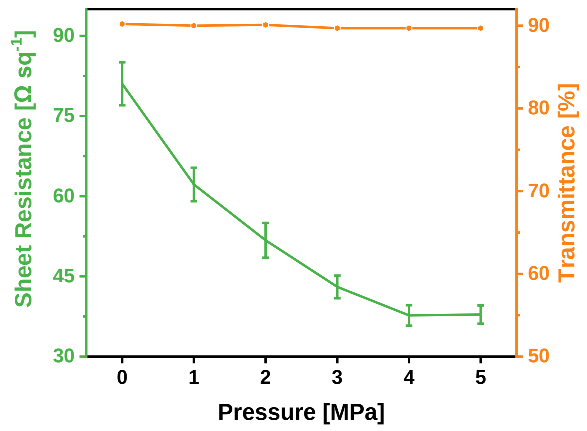

Figure S14. Effect of pressure on the sheet resistance and transmittance of the CuNWs electrodes.

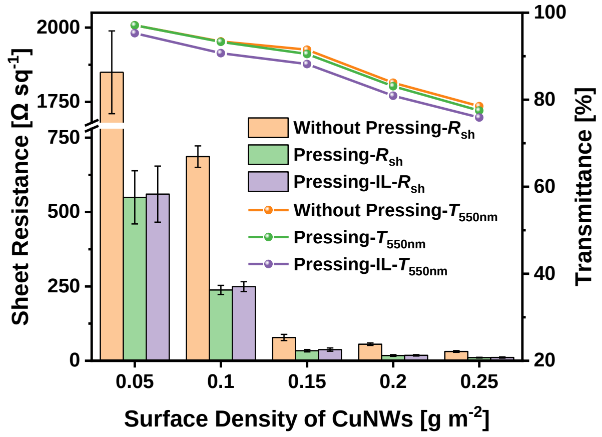

Figure S15. Sheet resistance and transmittance of the CuNWs electrodes for different surface densities of CuNWs before pressing, after pressing and after pressing followed by [BTAMMIM]TFSI treatment.

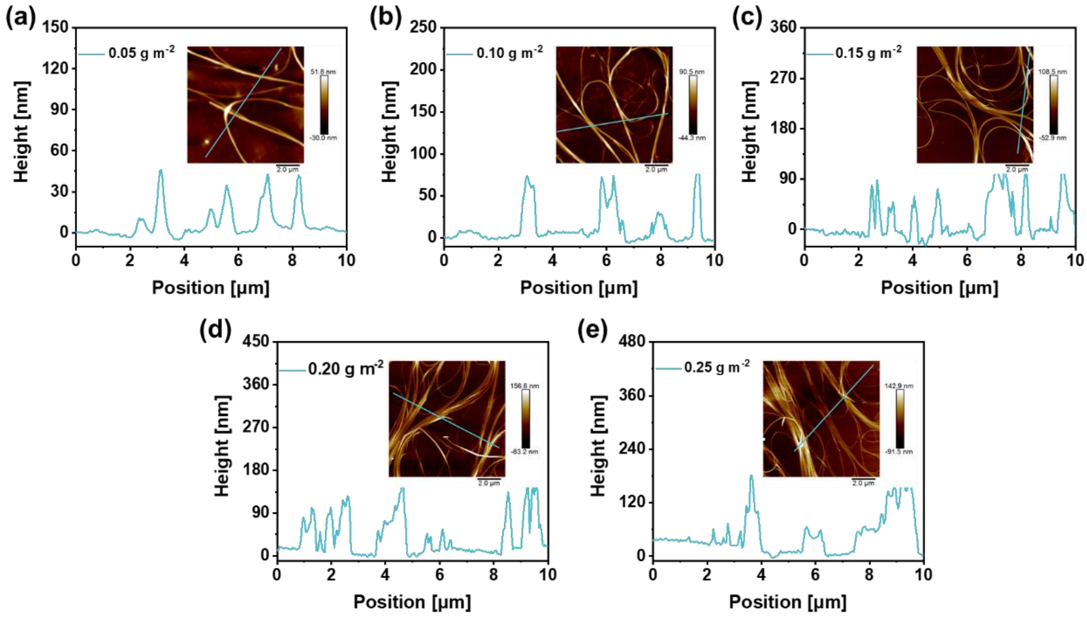

Figure S16. Height profiles of the CuNWs electrodes measured by scanning along the cyan line of the AFM images. Inset: The corresponding AFM images of the CuNWs electrodes for different surface densities of CuNWs.

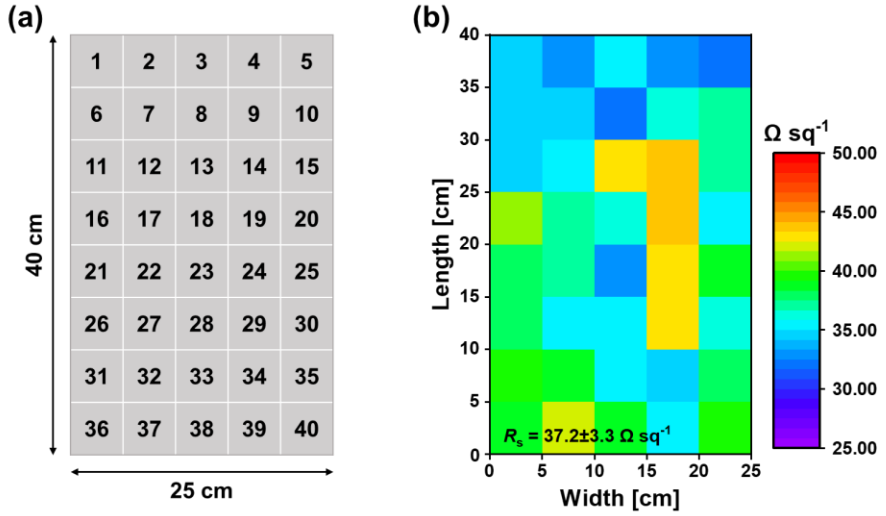

Figure S17. (a) Schematic illustration showing 5 × 8 pixels of the CuNWs@[BTAMMIM]TFSI composite electrodes for analysis. (b) Mapping images (5 × 8 pixels) showing the R_s distribution of the CuNWs@[BTAMMIM]TFSI composite electrodes.

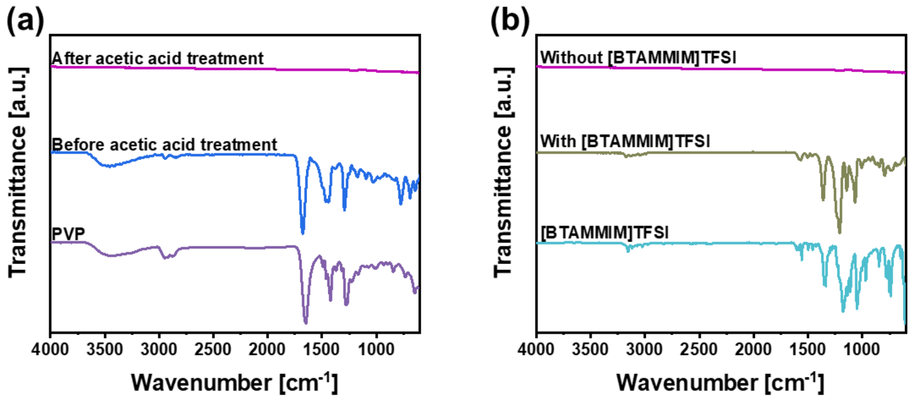

Figure S18. (a) ATR-FTIR spectra of PVP, CuNWs before and after acetic acid treatment. (b) ATR-FTIR spectra of [BTAMMIM]TFSI, CuNWs without and with [BTAMMIM]TFSI.

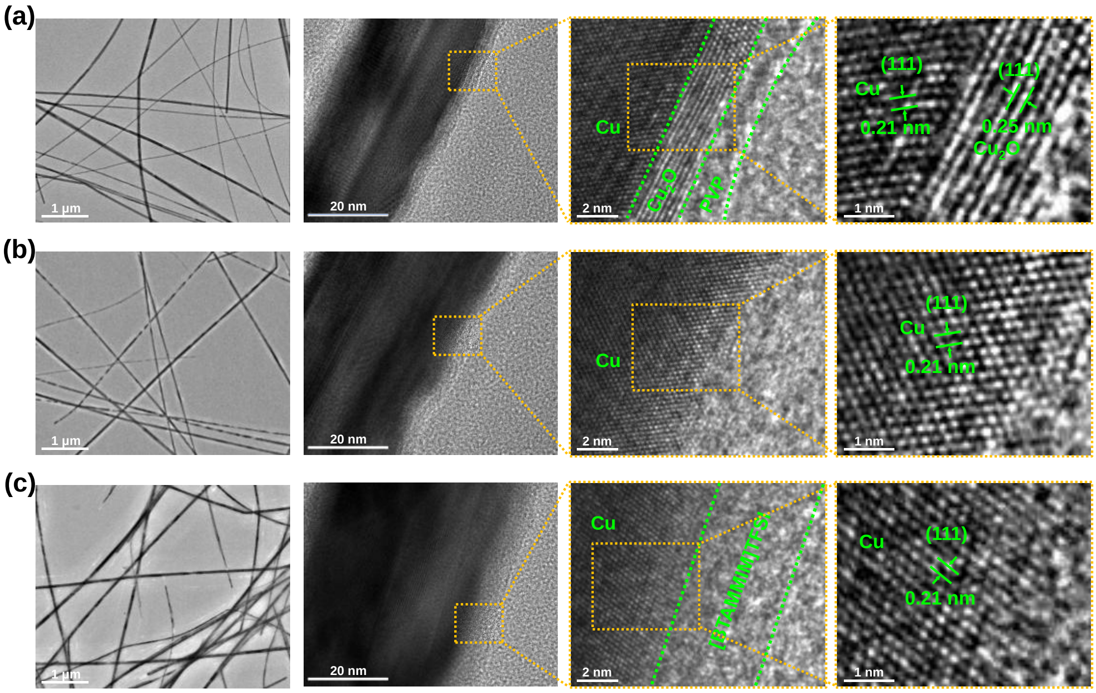

Figure S19. TEM and HRTEM images of CuNWs (a) before treating with acetic acid, (b) after treating with acetic acid and (c) after treating with acetic acid followed by [BTAMMIM]TFSI treatment.

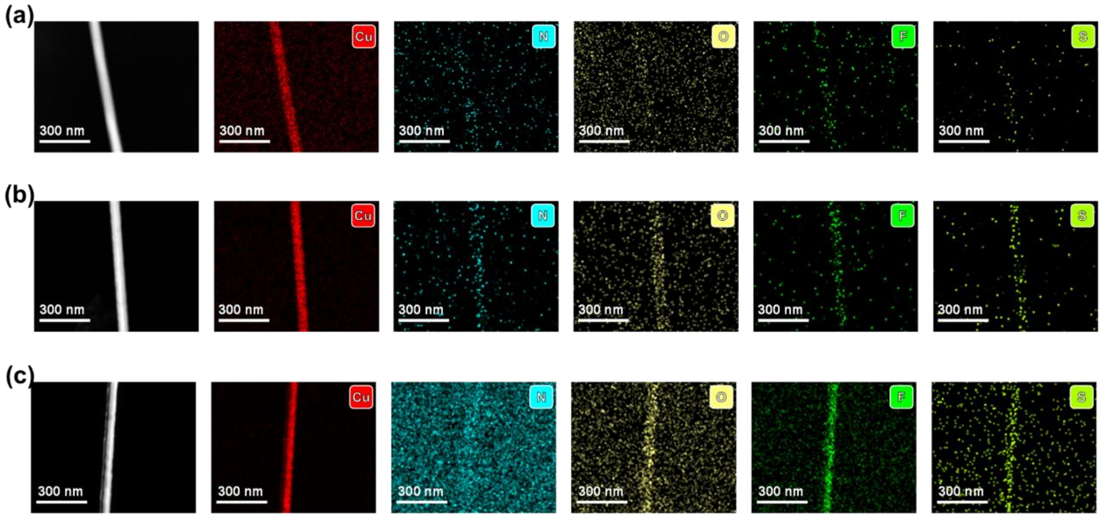

Figure S20. EDS elemental mapping images of CuNWs (a) before acetic acid treatment, (b) after acetic acid treatment and (c) after treating with acetic acid followed by [BTAMMIM]TFSI treatment.

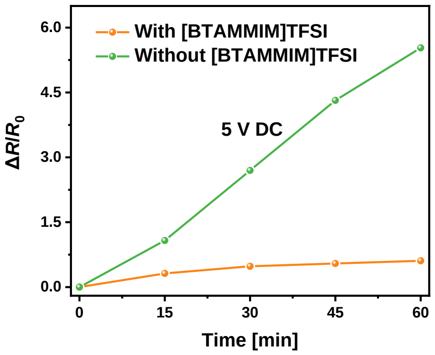

Figure S21. Relative changes in the sheet resistance of CuNWs-based electrodes with and without [BTAMMIM]TFSI under constant direct current (DC) voltage of 5 V.

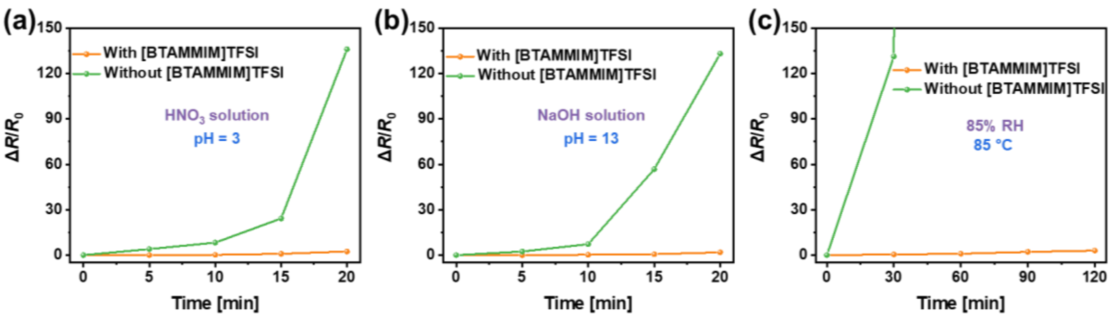

Figure S22. Relative changes in the sheet resistance of CuNWs-based electrodes with and without [BTAMMIM]TFSI in (a) acidic environment (HNO_3 solution, pH = 3), (b) basic environment (NaOH solution, pH = 13) and (c) a high temperature (85 °C) and high humidity (85% RH) environment.

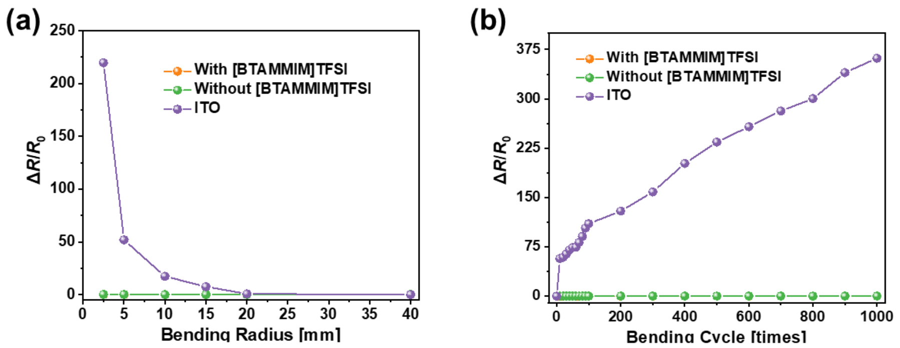

Figure S23. Relative changes in the sheet resistance of ITO film, CuNWs-based electrodes with and without [BTAMMIM]TFSI versus (a) bending radius and (b) bending times at a bending radius of 5 mm.

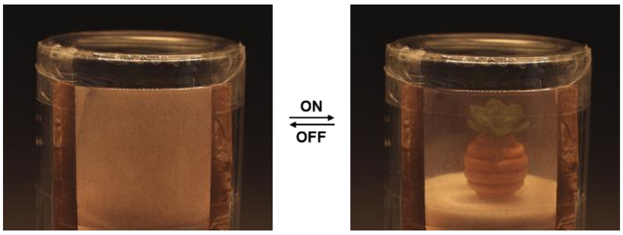

Figure S24. Optical images of CuNWs@[BTAMMIM]TFSI composite flexible transparent electrodes-based smart window fixed on a cylindrical glass to evaluate its potential applications in curved buildings.

References

[1] T.-H. Duong, H.-C. Kim, "Extremely Simple and Rapid Fabrication of Flexible Transparent Electrodes Using Ultralong Copper Nanowires" *Ind. Eng. Chem. Res.* **2018**, *57*, 3076-3082.

[2] M. Mohl, P. Pusztai, A. Kukovecz, Z. Konya, J. Kukkola, K. Kordas, R. Vajtai, P. M. Ajayan, "Low-Temperature Large-Scale Synthesis and Electrical Testing of Ultralong Copper Nanowires" *Langmuir* **2010**, *26*, 16496-16502.

[3] M. Cai, Y. Liang, F. Zhou, W. Liu, "Functional Ionic Gels Formed by Supramolecular Assembly of a Novel Low Molecular Weight Anticorrosive/Antioxidative Gelator" *J. Mater. Chem.* **2011**, *21*, 13399-13405.

[4] A. I. Rusanov, V. A. Prokhorov, *Interfacial Tensiometry, Vol. 3*, Elsevier, **1996**.

[5] Z. Shen, R. L. Mernaugh, H. Yan, L. Yu, Y. Zhang, X. Zeng, "Engineered Recombinant Single-Chain Fragment Variable Antibody for Immunosensors" *Anal. Chem.* **2005**, *77*, 6834-6842.

[6] H. Liu, Y. Li, K. Sun, J. Fan, P. Zhang, J. Meng, S. Wang, L. Jiang, "Dual-Responsive Surfaces Modified with Phenylboronic Acid-Containing Polymer Brush To Reversibly Capture and Release Cancer Cells" *J. Am. Chem. Soc.* **2013**, *135*, 7603-7609.

[7] D. A. Buttry, M. D. Ward, "Measurement of Interfacial Processes at Electrode Surfaces with the Electrochemical Quartz Crystal Microbalance" *Chem. Rev.* **1992**, *92*, 1355-1379.
